# All-perovskite tandem solar cells: from fundamentals to technological progress†

**DOI:** 10.1039/d3ee03638c

**Published:** 2024-05-10

**Authors:** Jaekeun Lim, Nam-Gyu Park, Sang Il Seok, Michael Saliba

**Affiliations:** a Institute for Photovoltaics (ipv), University of Stuttgart Stuttgart Germany michael.saliba@ipv.uni-stuttgart.de; b School of Chemical Engineering and Center for Antibonding Regulated Crystals, Sungkyunkwan University Suwon Republic of Korea npark@skku.edu; c SKKU Institute of Energy Science and Technology (SIEST), Sungkyunkwan University Suwon Republic of Korea; d Department of Energy Engineering, School of Energy and Chemical Engineering, Ulsan National Institute of Science and Technology Ulsan South Korea seoksi@unist.ac.kr; e Helmholtz Young Investigator Group FRONTRUNNER, IEK5-Photovoltaik, Forschungszentrum Jülich Jülich Germany

## Abstract

Organic–inorganic perovskite materials have gradually progressed from single-junction solar cells to tandem (double) or even multi-junction (triple-junction) solar cells as all-perovskite tandem solar cells (APTSCs). Perovskites have numerous advantages: (1) tunable optical bandgaps, (2) low-cost, *e.g. via* solution-processing, inexpensive precursors, and compatibility with many thin-film processing technologies, (3) scalability and lightweight, and (4) eco-friendliness related to low CO_2_ emission. However, APTSCs face challenges regarding stability caused by Sn^2+^ oxidation in narrow bandgap perovskites, low performance due to *V*_oc_ deficit in the wide bandgap range, non-standardisation of charge recombination layers, and challenging thin-film deposition as each layer must be nearly perfectly homogenous. Here, we discuss the fundamentals of APTSCs and technological progress in constructing each layer of the all-perovskite stacks. Furthermore, the theoretical power conversion efficiency (PCE) limitation of APTSCs is discussed using simulations.

## Abstract 2

This review provides fundamental knowledge and development directions towards the commercialising of advantageous all-perovskite tandem solar cells.

## Graphical abstract

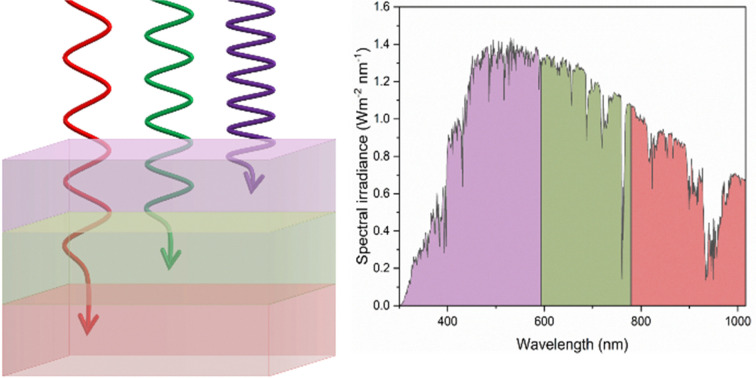

**Broader context**

Global warming caused by the excessive use of fossil fuels has accelerated the necessity for renewable energy sources. Metal halide perovskites are considered a promising next-generation technology for photovoltaics with rapid progress in performances in recent years and involve manufacturing processes with a low carbon footprint. All-perovskite tandem photovoltaics, constructed using multiple perovskite layers deposited on top of each other, are of particular interest because they permit more efficient use of available areas, require less consumption of materials and demonstrate an improved energy harvest. This is all the more compelling as recently all-perovskite tandems have exceeded the performances of both single-junction perovskite and silicon solar cells. In this review, we outline the theory and advantages of all-perovskite tandems as well as their potential to achieve even higher performances in the future.

## 1. Introduction

Organic–inorganic perovskites have shown great promise for photovoltaics (PVs). Perovskite single junction solar cells have been recently certified at >26% efficiency close to established silicon at >27% efficiency.^1^ Moreover, certified perovskite-based tandem solar cells have made improvements in a short period of time from 4.6% in 2014 to the current world record of 33.9%.^2,3^ Even perovskite–perovskite–silicon triple junction solar cells have been recently reported with an efficiency of 27.1%.^4^

**Fig. 1:**
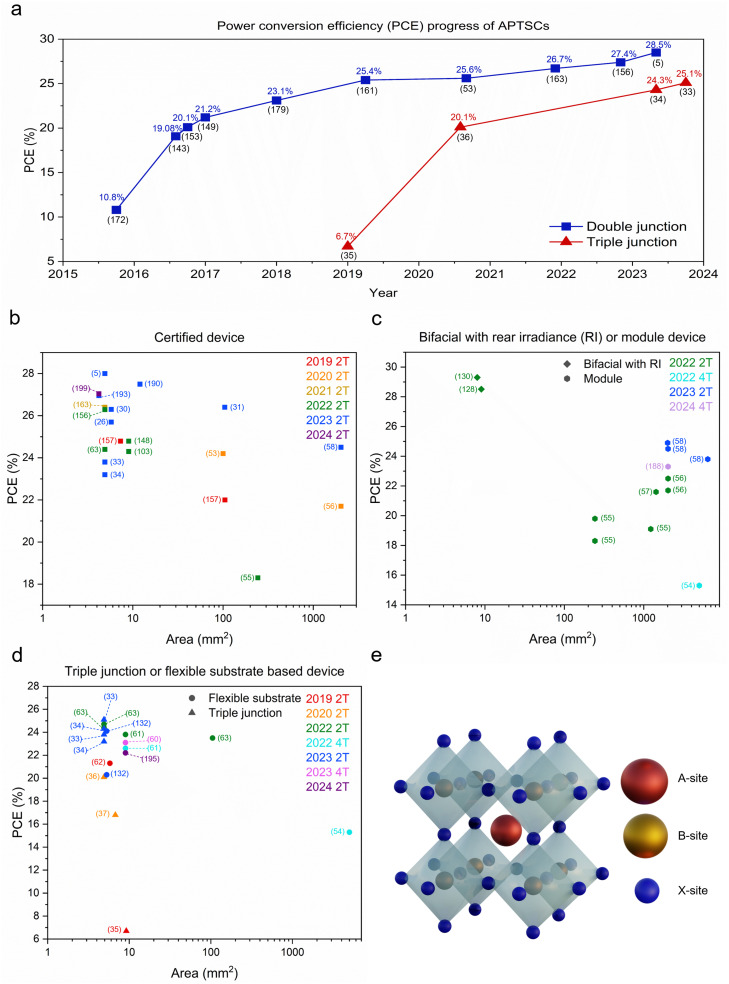
Summary graphs regarding efficiencies and active areas of APTSCs and the structure of perovskite. (a) This graph shows the power conversion efficiency progress of APTSCs regarding double and triple junctions with efficiency and according reference number just below (in brackets). The bifacial APTSCs are not included. (b) The scatter graph showing the efficiency (vertical axis) and area (horizontal axis) of APTSCs, regarding certified devices showing also the reference numbers. (c) The scatter graph showing the efficiency (vertical axis) and area (horizontal axis) of 2T and 4T APTSCs, containing the following information: bifacial and module device as well as the reference numbers. (d) The scatter graph showing the efficiency (vertical axis) and area (horizontal axis) of 2T and 4T APTSCs involving flexible substrate APTSC and triple junction APTSC information with the reference numbers. Device details (PCE, active area, terminal, and note) are in Table S1 (ESI†) regarding Fig. 1a–d. (e) The standard ABX_3_ perovskite structure.

Recently, APTSCs have been gaining attention with rapidly increasing performances, with 28.5% (certified: 28.0%) efficiency for a perovskite–perovskite tandem (double junction) (Fig. 1a–d).^5^ Although the efficiencies of APTSCs are still lower than silicon–perovskite tandems, they have several benefits. Here, we present the fundamentals of the APTSCs, providing the current research state as well as the future outlook.

Perovskites have remarkable material properties: direct bandgap, long diffusion length, long minority carrier life time, tunable bandgap and high defect tolerance.^6–10^ The ABX_3_ perovskite crystal structure comprises A, B, and X sites, where the A ion is positioned in the cuboctahedra vacancy formed by the X atoms in cuboctahedral AX_12_. The B site is occupied as an octahedron in a BX_6_ structure. The organic–inorganic metal halide perovskites are typically composed of (1) a monovalent A-site cation: caesium (Cs^+^), methylammonium (MA^+^) or formamidinium (FA^+^); (2) a divalent B-site metal: the lead(ii) (Pb^2+^) or tin(ii) (Sn^2+^) ion; and (3) a monovalent X-site anion: chloride (Cl^−^), bromide (Br^−^) or iodide (I^−^), superhalides or pseudohalides (Fig. 1e). The impact of the different components on the perovskite framework determines the motifs and properties of the perovskite crystal structure. For instance, large A-cations lead to 2-dimensional (2D) metal–organic sheets, which can alter electronic properties.^11–13^ Over time, some leitmotifs for perovskite were found, which predict the formability of the 3-dimensional (3D) organic–inorganic perovskite structure: the A-site component has a size constraint decided by the cuboctahedral vacancies created by the corner-sharing BX_6_ octahedra. The Goldschmidt tolerance factor (*t*) can be used to predict the stability of the 3D perovskite structure indirectly given by the ionic radii of elements A, B and X in the equation 
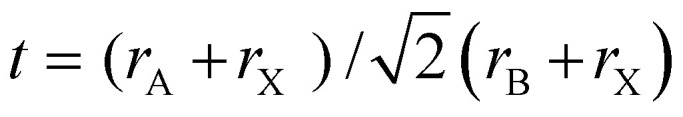
, where, *r*_A,B,X_ are the respective ionic radii.^14^

Experimentally, most 3D organic–inorganic perovskites have a Goldschmidt tolerance factor in the range of 0.80 ≤ *t* ≤ 1.00. If *t* > 1.00, the perovskites form a hexagonal structure. If *t* < 0.80, due to the small A cation, a non-perovskite structure is formed. A second restriction of the perovskite structure is the octahedral factor (*μ*), given by *μ* = *r*_B_/*r*_X_.^15^ Generally, in the range of 0.44 ≤ *μ* ≤ 0.90 a stable octahedral structure is formed.

The tolerance factor and octahedral factor inform about the formability of the perovskite structure. Much research concentrates on multi-component perovskites with any of the A, B, and X-sites rather than the pure structures of MAPbI_3_, FAPbI_3_, and CsPbI_3_, as they frequently suffer from phase instability (FAPbI_3_ and CsPbI_3_) or volatility (MAPbI_3_).

## 2. Advantages of APTSCs

### 2.1. High performance (tunability)

Tunable properties with compositional engineering boost research on perovskite solar cells (PSCs). In the organic–inorganic based perovskite structure (ABX_3_), the perovskite absorber layer from every A, B, and X position can modify the optical bandgap.^16–18^ For instance, in the A-site case, going from FA^+^, MA^+^ to Cs^+^ widens the bandgap (Fig. 3a). In the B-site case, generally, Sn^2+^ can create a narrower bandgap than Pb^2+^. At specific points of tin–lead mixed perovskite films, lower bandgaps than the pure tin-based perovskite film are observed.^19^ Lastly, in the X-site case, going from I^−^, Br^−^ to Cl^−^ widens the bandgap. Also, additive engineering with bulky organic molecules slightly changes the bandgap.^20–22^ The bandgap tunability of the perovskite solar cells (PSCs) allows to extend to the field of tandem technology.^23–26^ Especially, APTSCs have much potential for performance increase compared to single junction PSCs; however, dedicated bandgap alignment engineering is required. Recently, many studies have shown all-perovskite tandems (double junctions) with optimised narrow bandgaps between 1.21 and 1.26 eV and wide bandgaps between 1.73 and 1.78 eV with PCEs of 26.0–28.5%.^5,27–32^ On the other hand, the all-perovskite multi-junction bandgaps are not optimised yet for the highest performances, which will be a critical issue in the future.^33–37^

### 2.2. Low cost

Compared to conventional silicon solar cells, PSCs have the potential to be a competitive technology owing to: (1) low-temperature manufacturing, where most fabrication steps are below 150 °C, *e.g.*, in the so-called planar architecture that does not require high temperatures (since no mesoporous TiO_2_ is used). Although, compared with the conventional PV industry, even a 500 °C step (for mesoporous TiO_2_) could be considered as relatively low. (2) PSCs are a thin-film technology that need only a small amount of material.

**Fig. 2:**
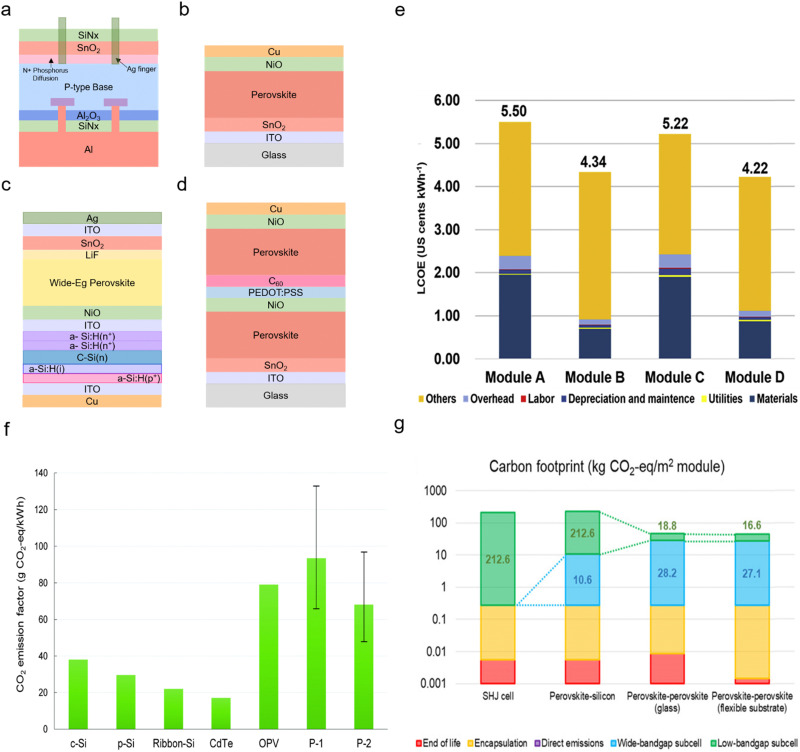
Low cost and low carbon footprint APTSCs: (a) conventional multi-crystalline silicon solar module. (b) The n–i–p planar structure of a perovskite single junction module. (c) The silicon/perovskite tandem solar module. (d) The perovskite/perovskite solar module. (e) Comparison of LCOE from figure (a)–(d). © 2018. Elsevier All rights reserved.^40^ (f) The past carbon footprint of c-Si, p-Si, ribbon-Si, CdTe, organic photovoltaic (OPV), and perovskite (P1: TiO_2_ based perovskite module, P2: ZnO based module). © 2018. Royal Society of Chemistry All rights reserved.^64^ (g) The recent carbon footprint of a silicon heterojunction (SHJ) cell, perovskite–silicon tandem (wide-bandgap perovskite: (Cs, FA)Pb(I, Br)_3_), perovskite–perovskite tandem with a glass substrate and perovskite–perovskite tandem with a flexible substrate consisting of a wide-bandgap (Cs, FA, MA)Pb(I, Br)_3_ and narrow-bandgap (FA, MA)(Sn, Pb)I_3_ perovskite. © 2020. American Association for the Advancement of Science All rights reserved.^66^

Furthermore, studies calculated the economic efficiency of perovskites at a relatively low cost with a levelized cost of energy (LCOE).^38,39^ For example, one study calculated the LCOE of two types of double junction and two types of single junction solar modules by adopting a bottom-up cost model to estimate the module cost (Fig. 2a–d). It claimed that the all-perovskite tandem solar module showed the most economical cost (Fig. 2e). All-perovskite tandem solar module showed the lowest LCOE with 4.22 US cents kWh^−1^ (Fig. 2d) compared to other modules (5.50, 4.34 and 5.22 US cents kWh^−1^; Fig. 2a–c).^40^ Another study calculated the LCOE of 2-terminal (2T) all perovskite tandem and 4-terminal (4T) all perovskite tandem PV modules. 2T all-perovskite tandem PV modules are slightly more competitive (US $33.8 per m^2^) than the 4T ones (US $42.3 per m^2^). However, the overall studies argued that perovskite solar cells have highly competitive prices.^41^

However, in the LCOE simulation, the stability of perovskite solar cells is a critical factor as longer lifetime devices mean lower LCOE values.^38–40,42^ In general, in the photovoltaic market, companies warrant the durability of, *e.g.*, 20 years with over 80% of the initial module PCE. Research of perovskite single junction solar cells demonstrated, *e.g.*, 4500 h of stability under illumination for PCEs >22% retaining more than 96% of the initial performance.^43^ Nonetheless PSCs (and thus APTSCs) still need more robust durability in the future to reach conventional PV module stability of 20 years with over 80% of the initial PCE.

The durability issues of APTSCs are more severe due to the different compositions of the perovskite absorbers. In the literature, most simulations for APTSCs assume long-term stability like conventional photovoltaics, which may be too optimistic for now although the durability of APTSCs is currently also rapidly developing. Hence, realistic LCOE simulations are essential. Furthermore, offering the LCOE results regarding outdoor-based APTSCs is important, as degradation rates can directly affect the LCOE output.

### 2.3. Scalable and lightweight

The scalability of solar cells is a critical issue for commercialisation escaping from the small lab scale. Many methods can be used for manufacturing large-area PSCs: blade coating, slot-die-coating, spray coating, inkjet printing, screen printing, and electro- and vapour-phase deposition.^44–50^ Spin coating is often used for lab scale fabrication, although there are examples of large-area PSCs using spin coating.^51^ Currently, it is challenging to determine which methods will be the frontrunner for commercialisation in the future since all fabrication strategies are developed in parallel. Moreover, most deposition techniques are rapidly advancing with better device performances, *e.g.*, blade coating reaching 23.2% (certified) at 7.3 mm^2^ in the cell and 18.2% at 3580 mm^2^ in the module.^52^ This scalability for the single-junction PSCs can also be extended to APTSCs similarly. Some studies demonstrated decent device performance with APTSCs in large areas. From 2016 to now, six research papers have been reported for large areas (>1000 mm^2^), including 2T and 4T. The large all-perovskite tandem solar modules' active areas reported in papers are 1200 mm^2^ (using a stack of glass/ITO/NiO/FA_0.8_Cs_0.2_Pb(I_0.6_Br_0.4_)_3_/C_60_/ALD-SnO_2_/Au/PEDOT-PSS/FA_0.7_MA_0.3_Pb_0.5_Sn_0.5_I_3_/C_60_/ALD-SnO_2_/Cu), 1225 mm^2^ (with a stack of MgF_2_/glass/IO:H/2PACz/wide bandgap perovskite/LiF/C_60_/SnO_*x*_/ITO and Au/PEDOT:PSS/narrow bandgap perovskite/PCBM/C_60_/BCP/Cu), 1430 mm^2^ (using a stack of glass/ITO/PTAA/wide bandgap perovskite/C_60_/SnO_2_/Au/PEDOT:PSS/narrow bandgap perovskite/PCBM/C_60_/BCP/Cu), 2025 mm^2^ (using a stack of glass/ITO/VNPB/NiO/Cs_0.35_FA_0.65_PbI_1.8_Br_1.2_/C_60_/ALD-SnO_2_/Au/PEDOT:PSS/FA_0.7_MA_0.3_Pb_0.5_Sn_0.5_I_3_/C_60_/ALD-SnO_2_/Ag), 2025 mm^2^ (using a stack of glass/ITO/NiO/VNPB/Me-4PACz/wide bandgap perovskite/C_60_/ALD-SnO_2_/Au/PEDOT:PSS/narrow bandgap perovskite/C_60_/ALD-SnO_2_/Cu) and 5000 mm^2^ (using a stack of wide bandgap: PET/ITO/SnO_2_/wide bandgap perovskite/Spiro-MeOTAD/VO_*x*_/IMI; of narrow bandgap: PET/ITO/PEDOT:PSS/narrow bandgap perovskite/C_60_/BCP/Cu), which demonstrated the PCEs of 21.4%, 19.1%, 21.6%, 22.5%, 24.5% and 15.3%, respectively.^53–58^ Except for the active area of 1200 mm^2^ (spin coating), all perovskite–perovskite tandem solar cells or modules were fabricated with blade coating.

Yet, the APTSCs still have particular challenges when using scalable fabrication methods, *e.g.*, when transferring optimised spin coating (lab scale) to the blade coating (industry scale) process. Firstly, the higher bromide concentrations in wide bandgap perovskite precursor solutions hinder high-quality, scalable fabrication due to the low solubility showing variable crystallisation kinetics. One study conducted by Xiao *et al.* attempted to tune the caesium concentration using a gas-assisted blade coating method, which resulted in enlarged average grain sizes from 290 to 380 nm.^56^ Secondly, it is challenging to eliminate excess perovskite precursor solvents such as DMF or DMSO after depositing perovskite layers due to low vapour pressure. The excess solvents disturb the formation of a uniform film and can be a challenge to process the narrow bandgap perovskite on top of the wide bandgap perovskite as those solvents can also dissolve the underlying wide bandgap perovskite layer. Fundamentally, selecting solvents with low boiling points can be achieved more easily than changing the blade coating parameters such as blade speed, the gap between the blade and the top layer or quenching gas pressure. Thirdly, both the top and bottom cells suffer from stability issues. Typically, Sn–Pb-based narrow bandgap perovskite bottom solar cells have a more critical stability challenge. In the future, large-area APTSCs may need to be manufactured in controlled atmospheres, which may be under nitrogen gas conditions or other controlled environments. Alternatively, the perovskite inks can be made more robust against outside variations. Finally, all perovskite tandem modules face challenges regarding module interconnection as described in Section 4.3 (Module interconnection of APTSCs). These reported papers indicated that APTSCs have the potential for commercial scale-up.^54,55^

Lightweight or flexible substrate-based architectures are another desired property in perovskite research. Flexible PSCs are a promising high-power-per-weight application.^59^ The flexible substrate-based APTSCs were reported in the papers using polyethylene naphthalate (PEN) or polyethylene terephthalate (PET) substrates with decent PCEs (Fig. 1d).^54,60–63^ These flexible or thin metal-based architectures may aid in developing economic roll-to-roll processing including an encapsulation process for APTSCs.^54^ However, all fabrication steps of APTSCs need to be checked for compatibility with roll-to-roll processing because of the speed and lower cost.

### 2.4. CO_2_ footprint (energy use)

The carbon footprint is the total amount of carbon dioxide along the entire value chain. At the beginning of the perovskite research, environmental research claimed that PSCs or organic solar cells have an issue with carbon dioxide (CO_2_) emission compared to other types of conventional photovoltaics such as Si-based solar cells or CdTe owing to a short lifetime (Fig. 2f). They considered the lifetime of the PSCs as approximately two years. Hence, the study mentioned that PSCs have 2–3 times higher carbon dioxide emissions than silicon photovoltaics.^64^ However, as perovskites have been quickly improving, recent research showed that <100 kg CO_2eq_ per kW_p_ for single-junction PSCs and <200 kg CO_2eq_ per kW_p_ for double junction APTSCs are among the lowest carbon dioxide emissions per kilowatt.^65^ Moreover, PSCs have the shortest energy payback time (EPBT) of 0.35 years among different types of solar cells. A study regarding the carbon dioxide footprint compared silicon/perovskite tandem solar modules and perovskite/perovskite tandem solar modules. It postulated a perovskite/silicon tandem with 25.2% efficiency and a perovskite/perovskite tandem with 23.1% efficiency as 2T architectures. Despite the perovskite/perovskite tandem modules having a lower efficiency than the perovskite/silicon tandem modules, carbon footprint calculations showed that perovskite/perovskite tandem modules emit less carbon than silicon heterojunction (SHJ) solar modules (212.6 kg CO_2eq_ per m^2^) or perovskite–silicon tandems (223.2 kg CO_2eq_ per m^2^) considering the lifetime of modules. When using the flexible substrate for perovskite/perovskite tandem solar modules (43.7 kg CO_2eq_ per m^2^), a slightly less carbon footprint was observed compared to a glass substrate (47.0 kg CO_2eq_ per m^2^) (Fig. 2g).^66^

## 3. Fundamentals of tandem solar cells

### 3.1. What are tandem solar cells?

Tandem (double junction) or multi-junction solar cells have been studied intensively over the years and are now a reliable technology, *e.g.* for space solar cells. The concept of tandem solar cells is to stack different absorber layers on top of each other so that each layer sequentially absorbs light close to its bandgap. Thus, the sunlight goes through the stack from the wide to the narrow bandgap material. This way, photons with energies lower than the bandgap are transmitted through the material. The photons with energies higher than the bandgap get absorbed while any excess energies beyond the bandgap are lost to internal thermal conversion.

The tandem solar cell avoids excessive losses in thermal energy by absorbing the higher energy photons in a suited material with a higher bandgap. Accordingly, architectures with two or three different absorber layers can be targeted adding complexity but also reducing the amount of heat loss.

### 3.2. Perovskite-based tandem or multi-junction solar cells

Ever since the concept of multi-junction solar cells was suggested in 1955, various tandem (double-junction) or multi-junction solar cells have been demonstrated to facilitate the development of highly efficient photovoltaics.^67^ For example, the highest efficiency of a multi-junction solar cell is 47.6% using optimised metal contacts and antireflection layers in a complex four-junction gallium indium phosphide (GaInP)/aluminium gallium arsenide (AlGaAs)/gallium indium arsenide phosphide (GaInAsP)/gallium indium arsenide (GaInAs) in a wide light spectral range between 300 and 1780 nm at a concentration of 665 suns.^68^

As the perovskite bandgap can be tuned from 1.2 to 3.0 eV, it can be flexibly employed with other absorber layers, such as perovskite/silicon solar cells, perovskite/organic solar cells, perovskite/copper indium gallium selenide (CIGS) solar cells, perovskite/dye-sensitized solar cells (DSSCs), perovskite/cadmium telluride (CdTe) solar cells and all-perovskite stacks.^23,24,69–71^

### 3.3. Theoretical performance limit of tandem solar cells

**Fig. 3:**
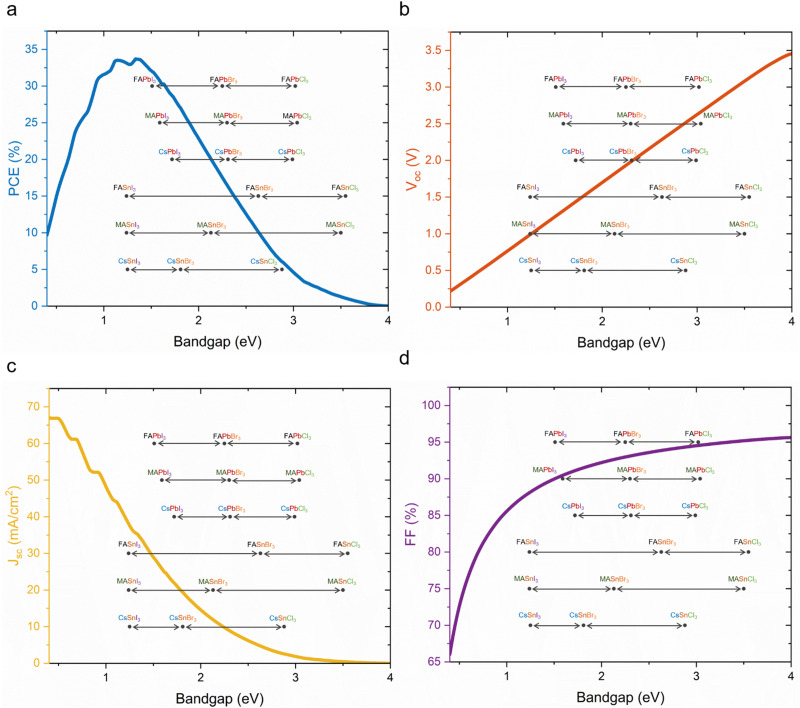
Prediction of single junction solar cells. (a) Shockley–Queisser limit graphs in terms of PCE, (b) *V*_oc_, (c) *J*_sc_ and (d) FF with the bandgaps of the different A-site, B-site and X-site perovskite compositions (the same colour indicates the same mono or polyatomic ions).

The Shockley–Queisser (S–Q) limit was calculated for the maximum efficiency in single junction solar cells considering only the radiative recombination loss while neglecting non-radiative recombination caused, *e.g.*, by Auger recombination and Shockley–Read–Hall recombination. Moreover, the S–Q limit assumes that each absorbed photon creates one electron/hole pair and that internal thermal conversion occurs, *i.e.*, photon energy in excess of the bandgap is lost as heat. Open-circuit voltage (*V*_oc_), short-circuit current (*J*_sc_) and fill factor (FF), which are factors of device efficiencies, show different trends with increased bandgap. *V*_oc_ increases linearly until a bandgap of 4 eV. *J*_sc_ decreases steeply, and around a bandgap of 3.5 eV reaches nearly 0. The FF can reach theoretically over 90% at 1.5 eV and a higher bandgap. According to the S–Q limit, the best possible theoretical efficiency is 33.16% with a bandgap of 1.34 eV under standardised air mass (AM) 1.5G illumination conditions (Fig. 3a–d).^72^

**Fig. 4:**
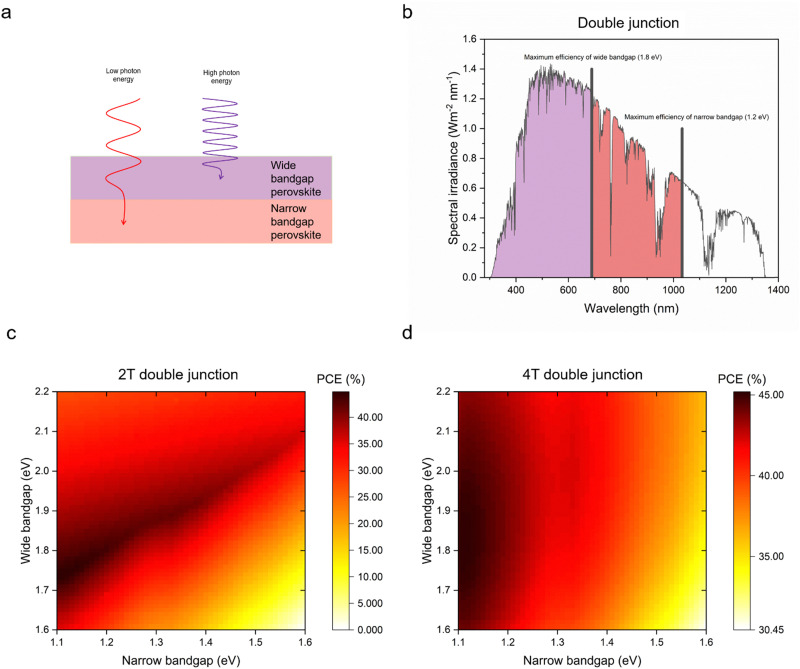
Prediction of double junction (tandem) solar cells: (a) scheme of photon energy absorption by wide and narrow bandgap perovskites in double junctions, (b) spectral irradiance graph showing theoretical maximum power conversion efficiency bandgaps for a 2-terminal (2T) double junction, (c) theoretical efficiency limit of tandem solar cells in 2T, which is a monolithic structure considering narrow bandgaps (1.1–1.6 eV) and wide bandgaps (1.6–2.2 eV), and (d) theoretical efficiency limit of tandem solar cells in 4-terminal (4T), which is a stacked structure mechanically considering narrow bandgaps (1.1–1.6 eV) and wide bandgaps (1.6–2.2 eV).

**Fig. 5:**
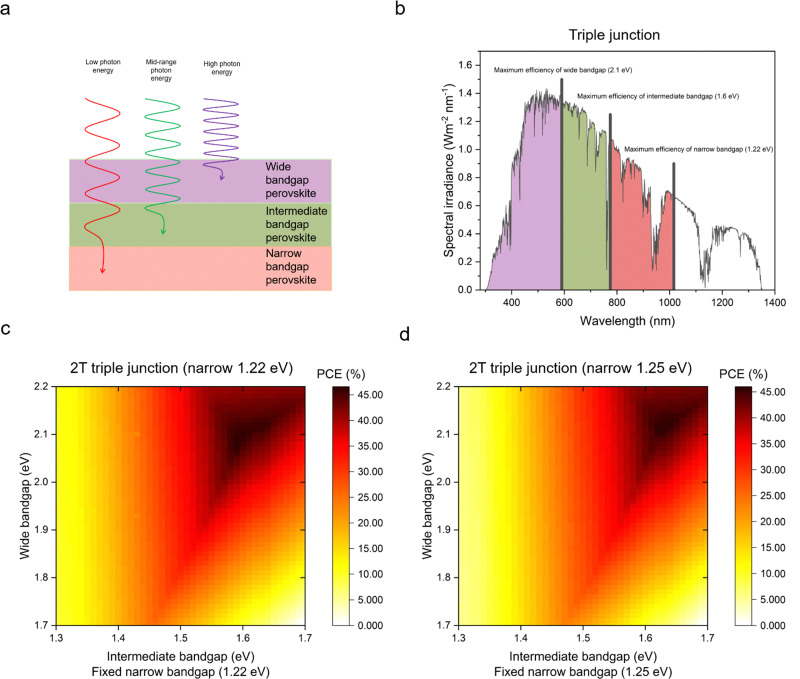
Prediction of triple junction (multi-junction) solar cells. (a) Scheme of photon energy absorption by wide and narrow bandgap perovskites in triple junctions, (b) spectral irradiance graph showing theoretical maximum power conversion efficiency bandgaps for a triple junction with a 1.22 eV narrow bandgap. (c) Theoretical efficiency limit of triple junction solar cells with a monolithic structure considering the narrow bandgap (the fixed value is 1.22 eV), intermediate bandgap (1.3–1.7 eV) and wide bandgap (1.7–2.2 eV), and (d) theoretical efficiency limit of triple junction solar cells in a monolithic architecture considering the narrow bandgap (a fixed value of 1.25 eV), intermediate bandgap (1.3–1.7 eV) and wide bandgap (1.7–2.2 eV).

For higher efficiencies, a tandem solar cell with multiple materials is required. Although the ideal bandgap for the wide bandgap perovskite is close to 1.7 eV for Si or CIGS bottom cells (bandgap 1.1 eV), a higher bandgap of around 1.8 eV is preferred to pair with the bottom narrow-bandgap (around 1.2–1.3 eV) perovskite solar cells.^24^ The highest efficiencies that can be achieved with double junction tandems are 46% and 46.1% for 2T and 4T considering bandgaps from 0.5–2.5 eV, respectively.^73^ However, achieving perovskite bandgaps <1 eV, needed for high multi-junction stacks, is challenging. All perovskite tandem solar cell studies are restricted to the narrowest perovskite bandgap at 1.2 eV (using mixtures of Sn and Pb).^19^ Many studies employed a well-optimised narrow bandgap of 1.22 eV. Therefore, utilising a top cell with 1.82 eV and a bottom cell with 1.22 eV enables a theoretical 43% PCE for a 2T tandem, which is similar to a 4T tandem, where the bandgap of the top cell can range from 1.75 to 2.08 eV, under AM 1.5G illumination as shown in Fig. 4a–d. So far, 1.22 eV and 1.25 eV as bottom bandgaps were used for triple junction all perovskite solar cells. In the theoretical PCE limit calculation, when a bottom bandgap of 1.22 eV was employed, an intermediate bandgap of 1.59–1.61 eV and a wide bandgap of 2.08–2.11 eV combination can achieve over 46% PCE. When a bottom bandgap of 1.25 eV was used, an intermediate bandgap of 1.62–1.65 eV and a wide bandgap of 2.1–2.13 eV combination can theoretically achieve over 45% PCE, under AM 1.5G illumination as shown in Fig. 5a–d. Although this calculation does not include the thickness of absorber layers, recent APTSC research showed that around 400 nm is desired for a wide bandgap, over 1000 nm for a narrow bandgap in a double junction and approximately 300 nm for a wide bandgap and over 1000 nm for intermediate and narrow bandgaps in a triple junction as shown in Table S2A and B (ESI†). Many works have optimised the thicknesses of double-junction perovskite absorber layers. However, there is not sufficient research yet on the optimised thicknesses for triple junction APTSCs. This review provides APTSC simulation information regarding potential efficiency in Section 7 and Table 3 considering the electrical properties of real devices' stacks.

### 3.4. Wide bandgap perovskite solar cells

Wide bandgap perovskite solar cells had already been successfully integrated with narrow-bandgap absorbers such as Si (1.12 eV), CIGS (1.1 eV), and tin (Sn)–lead (Pb)-based-perovskites (1.25 eV), resulting in tandem architectures with promising efficiencies of 33.9%, 24.2%, and 28.5%, respectively.^5,24^

However, unlike well-investigated perovskites with bandgaps of 1.4–1.6 eV for single junction or narrow bandgap perovskites (1.2–1.4 eV), wide bandgap perovskites have a relatively large *V*_oc_ deficit, *e.g.*, a *V*_oc_ of 1.7 V (theoretical limit is 1.98 V) for a bandgap of 2.3 eV contrasting narrow bandgap perovskites with 1.26 V (theoretical limit is 1.32 V) for a bandgap of 1.6 eV.^74,75^ The *V*_oc_ is a key performance indicator towards higher efficiency of APTSCs.

Especially, 2T APTSCs have a significant opportunity to improve efficiencies as the total *V*_oc_ in the series connection is the sum of the individual subcell's *V*_oc_. In a study, FACs-based (1.77 eV bandgap) PSCs were tested to determine the cause of the *V*_oc_ deficit. Although the theoretical *V*_oc_ is 1.49 V at 1.77 eV, the device showed 1.03 V. It was concluded that trap-assisted, non-radiative recombination (∼400 mV) and photoinduced phase segregation (∼100 mV) reduce the *V*_oc_. The main reason for the *V*_oc_ deficit is the non-radiative recombination within the perovskite absorber and interface between perovskite and charge extraction layers such as electron transport layers or hole transport layers.^76^ Up to now, using new materials for the charge extraction layer, additive engineering in perovskite precursors and passivation layers between the perovskite absorber layer and charge extraction layer have been applied to reduce non-radiative recombination.^77–81^

Here, the wide bandgap perovskites are discussed within the context of inorganic perovskites and organic–inorganic hybrid perovskites with mixed halides and cations.

#### 3.4.1. Inorganic perovskite solar cells

Incorporating fully Cs^+^ in a perovskite crystal as an A-site instead of MA^+^ or FA^+^ organic molecules increases thermal stability.^82–86^ CsPbX_3_-based (X: halides) structures have bandgaps from 1.72 eV to 2.99 eV (Fig. 3a).^17^ As a side note, inorganic perovskites may be suited for space applications due to thermal stability at temperatures >200 °C.^87^

However, in inorganic perovskites, stability challenges arise when devices are exposed to humidity, oxygen and illumination conditions.^88^ The most extensively studied inorganic perovskites are CsPbI_3_, CsPbBr_3_, and halide mixtures thereof CsPbI_3−*x*_Br_*x*_, where *x* is 0 < *x* < 3.

CsPbI_3_ has four different types of phases, one photoinactive yellow phase, which is the orthorhombic δ-phase, and three photoactive black phases: cubic phase (α-phase), tetragonal phase (β-phase), and orthorhombic phase (γ- phase). α-phase CsPbI_3_ has the ideal bandgap (1.73 eV). To convert from the δ-phase of CsPbI_3_ to the α-phase, a high temperature of 360 °C is needed. In the process of cooling after annealing, CsPbI_3_ transforms into the original yellow δ-phase. However, in the process of phase change, CsPbI_3_ shows a series of metastable black polymorphs. The symmetry α-phase begins to transit to the β-phase at 260 °C, followed by a further loss of symmetry to the γ-phase at 175 °C. During the α → β → γ transition, the high symmetry is removed because of octahedral tilting and macroscopic strains. Consequently, stable CsPb(I_1−*x*_Br_*x*_)_3_-based solar cells were demonstrated with various deposition techniques and surface or additive engineering. Simultaneously, this inorganic perovskite-based wide bandgap APTSC showed relatively high performance with 25.6% PCE.^89^

#### 3.4.2. Mixed halide or cation-based wide bandgap solar cells

Not only Cs-based perovskite films but also standard organic cation-based perovskite films can contribute to tandem solar cells. They are favourable due to their wide bandgaps utilising mixed halide. The crystallisation of a mixed halide perovskite with Br^−^ and I^−^ (MA_0.9_Cs_0.1_Pb(I_0.6_Br_0.4_)_4_) can achieve a bandgap of 1.8 eV following the Ostwald ripening crystal growth.^90^ Another example demonstrated reaching a wide bandgap ultimately under Cs^+^ free conditions and modifying only the halide part, *i.e*., MAPbI_*x*_Br_3−*x*_. The composition allows the optical bandgap to reach around 1.70–1.75 eV.^91,92^

However, the I–Br mixed halide perovskites have shown phase segregation resulting in different bandgap energies.^93,94^ This photoinduced phase segregation forms the I-rich and Br-rich domains on surfaces and grain boundaries under illumination resulting from ion migration.^95^ This phenomenon can affect the production of photoinduced traps, reduction of charge mobility, and short carrier lifetime causing *V*_oc_ loss.

Hence, typically Cs_*x*_FA_1−*x*_PbI_3_ (*x* is from 0 to 1) perovskites have been investigated to avoid the halide segregation problem.^96^ After photoinduced segregation, a self-healing phenomenon can be observed to recover from the damage of two-photon microscopy.^97^ When Cs is >0.5 in Cs_*x*_FA_1−*x*_PbI_3_, a potentially higher stability than pure CsPbI_3_ was observed due to a more appropriate tolerance factor. However, because of the different phase transition temperatures between FAPbI_3_ and CsPbI_3_ perovskites, and the large lattice mismatch of perovskite structure, the quest for phase stable materials without segregation is still ongoing.^98^

### 3.5. Narrow bandgap PSCs

The development of narrow bandgap PSCs is also one of the main factors responsible for the compatible APTSCs replacing the silicon or CIGS bottom cells. In recent research, tin-based PSCs or tin–lead mixed PSCs provided promising results.^99–102^ Partial substitution of Sn into Pb perovskites forming the mixed Sn–Pb perovskites can tune the bandgap to the near-infrared region (1.2 to 1.4 eV), which can be employed for ideal bandgap single-junction PSCs or be paired with wide bandgap PSCs to fabricate APTSCs.^16^ The mixed Sn–Pb perovskites possessed unique optoelectronic properties, such as high conductivity, likely due to high background carrier concentrations, and the bandgap bowing effect (not following the Vegard law) that showed non-linearity of the bandgap graphs.^103,104^ This shows that Sn–Pb perovskites (Pb_0.25_Sn_0.75_ alloy) have a lower bandgap than pure Sn perovskite.^105^ Moreover, Sn or Sn–Pb perovskites have been developed for fabricating low-toxicity PSCs.^106–108^

#### 3.5.1. Pure Sn (lead-free)-PSCs

A pure Sn-based perovskite absorber layer is in the spotlight due to the broad absorption range with a bandgap of 1.24 eV, thus including the infrared region, high charge mobilities, long carrier diffusion, and lifetimes.^109,110^ MA^+^ is hygroscopic.^111,112^ Hence, MA^+^ can react with water caused by the degradation of MASnI_3_ perovskite devices. Sn–O bonds are produced between water molecules and the surface Sn atoms, breaking Sn–I bonds with the I atoms of the closet MAI layer.^113^ Therefore, pure Sn-perovskites suffer from instability due to the Sn^2+^ to Sn^4+^ oxidation, leading to the defect formation of Sn vacancies in the crystal structure and resulting in high p-doping.^114^ The tin vacancies act as the main recombination sites for charge carriers causing much-shortened durability and poor operational stability of 500 h (under 75% relative humidity conditions) compared with their wider bandgap (>1.5 eV) counterparts, which are, *e.g.*, stable for 1200 h (at 85 °C and 85% relative humidity) retaining 86% of the initial efficiency.^115,116^ This imposes challenges for the monolithic APTSC structure.^117^

#### 3.5.2. Sn–Pb mixed PSCs

MASnI_3_ perovskites did not demonstrate significant photovoltaic properties for a long time because of the unstable Sn^2+^. Hence, a small content of Pb is often added to fabricate PSCs and stabilise Sn^2+^. The alloyed perovskite with MASnI_3_ and lead analogue attracted interest as a narrow bandgap absorber exhibiting 1.2–1.3 eV for single-junctions and the bottom cells for APTSCs. The PCEs of Sn–Pb mixed-perovskites have improved from 7.37% for MASn_0.25_Pb_0.75_I_3_ at 1.24 eV in 2014 to 23.2% for MASn_*x*_Pb_1−*x*_I_3_ (*x* is from 0 to 1) at 1.21 eV in 2022.^16,118^ Sn–Pb-mixed perovskites have exhibited a better stability than pure Sn-based perovskites due to reduced (unwanted) tin oxidation, improving film quality and binding energies. Nevertheless, the stability issue still remains a challenge for Sn-containing perovskites.^119^ One facile solution is compositional engineering. Instead of the pure MA^+^, adding FA^+^ or Cs^+^ improved the efficiency to 21% and 1000 h of thermal stability under air and dark conditions at 85 °C without encapsulation.^120,121^ Currently, Pb–Sn-mixed-perovskite is the only viable narrow bandgap absorber and thus, the only option for the bottom cell in APTSCs. Therefore, many methods were used for studying the Sn^2+^ instability, which thus will be elaborated below.

## 4. Architectures of APTSCs

Each configuration of the tandem solar cells has advantages and disadvantages. APTSCs have a similar architecture to silicon/perovskite tandem solar cells.^122^ Commonly, 2T and 4T were demonstrated in many studies.

### 4.1. 2T APTSCs

**Fig. 6:**
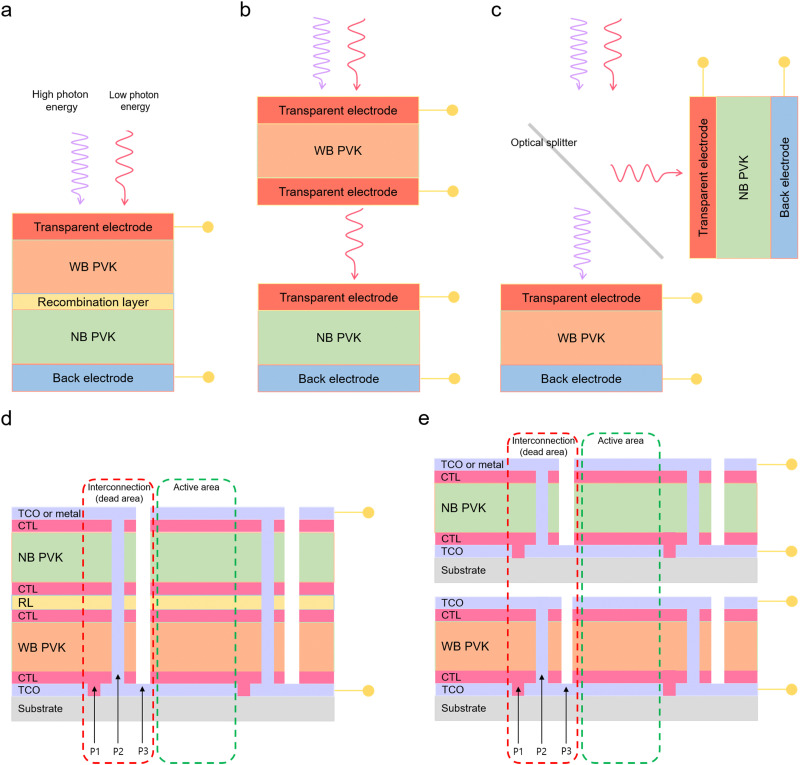
Standard double junction configuration of tandem solar cells and configuration of all perovskite tandem modules. (a) 2-Terminal (2T) configuration. (b) 4-Terminal (4T) mechanically stacked, (c) and 4-terminal-optically splitting configuration. The optical splitter separates the high photon energies and low photon energies. High photon energies are absorbed by wide bandgap perovskite layers and low photon energies are absorbed by narrow bandgap perovskite layers. © 2021. John Wiley and Sons All rights reserved.^127^ (d) Scheme of 2-terminal all-perovskite-based tandem module configuration with interconnection (dead area indicated by red dashed lines) consisting of patterns 1, 2 and 3 (P1, P2 and P3) and active area (green dashed lines). (e) Scheme of 4-terminal all-perovskite-based tandem module configuration. TCO is the transparent conductive oxide. CTL is the charge transport layer. WB PVK is the wide bandgap perovskite layer. RL is the recombination layer.

The concept of 2T APTSCs is to fabricate all the layers on the same substrates, *i.e.* a monolithic architecture. The stack follows back electrode/narrow-bandgap perovskite (bottom cell)/interconnecting layer (charge recombination layer)/wide-bandgap perovskite (top cell)/transparent electrode (Fig. 6a). Thus, 2T tandems only need one substrate, which is one of the cost advantages. However, when the top layers of the overall tandem are deposited, it is crucial not to damage the underlying layers, *e.g.*, by solvent addition or sputtering. In addition, the configuration of the interconnecting layers is important. Three different interconnection viewpoints should be considered to create the high-quality interconnecting layers: electrical, optical and mechanical. Electrical interconnection is considered to extract carriers from the near subcells and to facilitate the recombination in the subcells. Optical interconnection needs to address optical transparency issues to reduce absorption losses. Mechanical interconnection is required in order not to damage the underneath subcells during the device fabrication.^123^ This review describes the main strategies for manufacturing interconnecting layers in Section 6.3.

### 4.2. 4T APTSCs

4T tandems use two different substrates (Fig. 6b and c). Moreover, 4T APTSCs do not require current matching as there is no series interconnection mechanically. Thus, the fabrication process of 4T tandems is more convenient than that of 2T tandems, although it employs more materials and is prone to higher optical losses from parasitic absorption and unwanted reflection. 4T tandems have two types of designs. One type is similar to 2T but requires four electrodes. However, the used area is the same as 2T tandems. Another type is an installed optical splitter between two subcells, which are located perpendicularly. Therefore, one subcell absorbs high energies of photons, another absorbs low energies of photons. This method can be more efficient when it comes to large areas for commercialisation, although the additional costs for the optical splitter needs to be factored in as well.^123^

### 4.3. Module interconnection of APTSCs

Perovskite solar modules are commonly manufactured by an integrated series interconnection of subcells. Fig. 6d and e show the scheme of 2T and 4T all-perovskite tandem modules, respectively. For fabricating all-perovskite modules, a scribing process with three patterning steps P1, P2 and P3 is used:

P1 scribing is carried out on a transparent conductive oxide (TCO) based substrate. P2 scribing is implemented before depositing the last conductive layers, which means removing all layers except the bottom TCO. These P2 lines interconnect the subcells. After the top electrode deposition, such as TCO or metal, P3 lines are scribed to isolate the top electrode from adjacent cells. Although this patterning process is necessary, it comes with drawbacks, such as dead areas where the scribing was applied (not creating electricity). Therefore, the dead areas must be minimised without producing shunting losses. A study demonstrated how the subcell width can affect the performance of all-perovskite tandem modules. Increasing the subcell width (6.4–15.0 mm) resulted in having higher geometric fill factors (88.3–95.0%) and instantly affected higher module efficiencies (the highest efficiency is 21.2% with a width of 11.25 mm and a geometric fill factor of 93.3%). However, from the width of 7.5 mm, series resistance increased, and consequently, the fill factor of the module was reduced following a parabolic shape.

After the P3 lines, each small cell is exposed to ambient atmospheres such as oxygen or moisture causing degradation. In perovskite tandem modules, halides from the perovskite absorbers can react with the metal electrodes at the interconnecting subcells. This may create deep defects, which can be mitigated using, *e.g.*, a thin conformal diffusion barrier deposited, *e.g.*, by atomic layer deposition.^56^ As a result, the conformal diffusion barrier hampered halide–metal interdiffusion avoiding the reaction between the perovskite layer and the metal electrodes improving stability. Finally, 4T all-perovskite tandem modules consist of two submodules. Hence, it is important to align the patterned areas carefully to prevent power losses.^54,55,57,124–126^

## 5. More specific APTSCs

Less standard configurations use bifacial APTSCs, inorganic-based APTSCs, flexible APTSCs, substrate configured or multi-junction designs.

### 5.1. Bifacial all-perovskite tandems

**Fig. 7:**
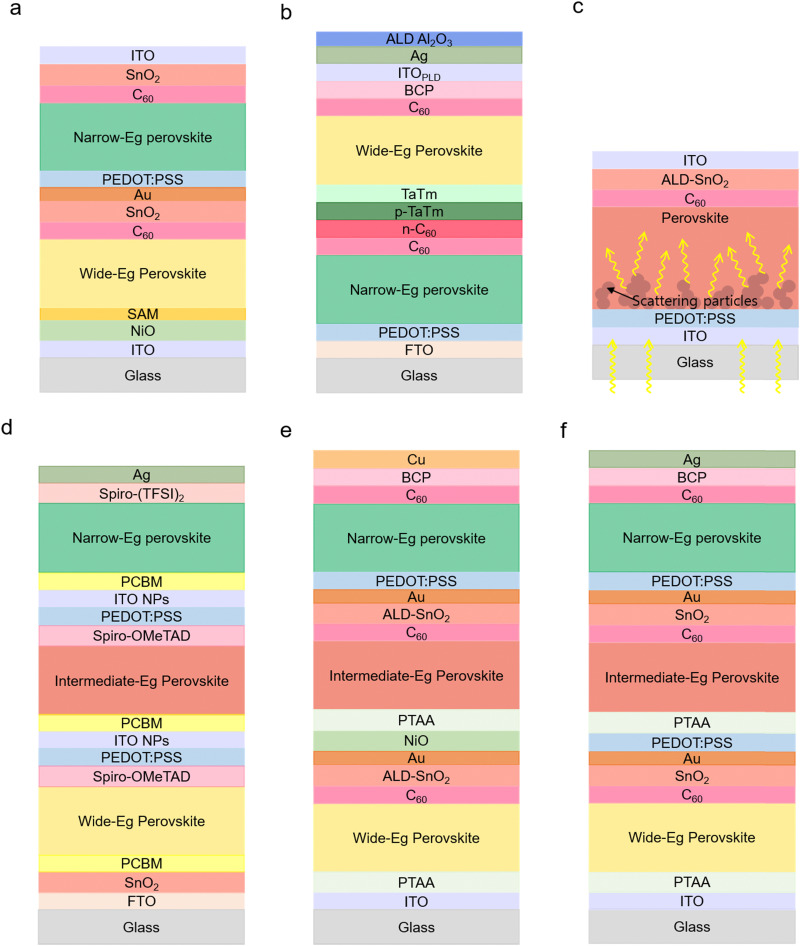
Various types of APTSCs. (a) Scheme of the bifacial APTSC configuration. © 2022. Springer Nature All rights reserved.^128^ (b) Another type of bifacial APTSC configuration. © 2022. American Chemical Society All rights reserved.^129^ (c) Illustration of the scattering particles (resin) functioning with increasing photon path length in the narrow bandgap perovskite film. © 2022. American Association for the Advancement of Science All rights reserved.^130^ (d) The APTSC structure of a fully solution-processed triple junction.^35^ (e) (f) Other types of solution-processed triple junction APTSC configurations. © 2020. American Chemical Society All rights reserved. © 2020. Springer Nature All rights reserved.^36,37^

A bifacial solar cell generates more electricity using light from the front and rear side. The bifacial APTSCs were reported in various studies in the literature: Li *et al.* suggested the initial design of bifacial APTSCs using TCO as the back electrode (Fig. 7a). Moreover, the optimised current matching design for mono-facial APTSCs resulted in the current mismatch in the bifacial configuration because the current of the bottom side will be increased with albedo in bifacial monolithic tandems. Besides, the back side TCO led to a reduced current as there was no light reflection parts like metals to guide more light into the perovskite absorber. For example, at the wide bandgap of 1.77 eV, mono-facial APTSCs showed 24.36% and bifacial APTSCs showed 23.34% and 25.25% without and with 30 mW cm^−2^ rear irradiance (RI) conditions respectively. Although under the RI conditions, a 1.68 eV bandgap indicated the highest efficiency of 28.51%, without RI, only 19.29% efficiency was obtained.^128^ Another study also focused on creating TCO electrodes for both sides, demonstrating a PCE of 20% (Fig. 7b).^129^ Chen *et al.* implemented several techniques for improving energy yield: by decreasing the optical bandgap of a wide bandgap cell to 1.65 eV instead of 1.78 eV and by specially embedding a light-scattering micrometre-sized particle layer into a narrow bandgap perovskite (Fig. 7c). As the embedding particles, the cation-exchange resin particles were employed to obtain the light-scattering function to lock the Pb in perovskite devices, which is important for leakage cases. This particle layer supported solving the removal of the reflection back metal electrode and the reduction in the photon path length. This method effectively absorbed 5 to 15% in the infrared region. The highest efficiency of a bifacial APTSC was obtained at a wide bandgap of 1.65 eV with 29.3% under the 30% albedo light conditions. However, without albedo light conditions, the bandgap of 1.65 eV (16.7%) led to a lower efficiency than the bandgap of 1.78 eV (20.6%).^130^

### 5.2. Inorganic APTSCs

As mentioned above, inorganic perovskites have a favourable optical bandgap (∼1.77 eV) for wide bandgap solar cells and high thermal stability. Until now, one publication demonstrated APTSC with an inorganic wide bandgap subcell. However, the interface between the transport layer and inorganic perovskite has challenges of a significant energetic mismatch and limited charge extraction. To solve this, interfacial engineering was applied.^131^ Furthermore, a study demonstrated fully inorganic-based APTSCs in a 4T integration.^232^

### 5.3. Flexible APTSCs

Flexible APTSCs provide the opportunity for mass production by a roll-to-roll process in the future. Currently, various works use a flexible substrate, such as polyethylene naphthalate (PEN) or polyethylene terephthalate (PET), although the loss of open-circuit voltage at a wide bandgap is more distinct than that on rigid substrates. Another challenge is depositing layers uniformly on the flexible substrate since flexible substrates typically have rougher surfaces than the rigid ones. To resolve these issues, primarily interfacial layer engineering or additive engineering was implemented. These flexible substrate-based APTSCs were tested on the 4T and 2T configurations, *e.g.*, with the largest area being at 5000 mm^2^ on a PET substrate with a PCE of 15.3% in 4T APTSCs. The highest efficiency on a flexible PET substrate with a 4.9 mm^2^ active area was 24.7% in 2T APTSCs.^54,60–63^

### 5.4. Substrate-configured APTSCs

The generally used superstrate configuration, where the wide bandgap subcell comes first followed by the narrow bandgap subcell, has stability challenges in the fabrication. Especially, the narrow bandgap perovskite comprises tin–lead mixtures, which are particularly exposed to the oxygen environment, to which they are more sensitive compared to the wide-bandgap perovskite at the bottom. A substrate-configured device is fabricated by depositing the first narrow-bandgap perovskite and subsequently the wide-bandgap perovskite. Hence, the narrow bandgap perovskite embedded at the bottom is more protected from oxygen conditions. This can improve oxidation resistance and thus stability. Furthermore, this configuration can utilise all substrates flexibly, from transparent substrates such as glass, PET, PEN, and polyimide (PI) to opaque substrates like polymers, stainless steel or metal foils.^132^

### 5.5. Multi-junction APTSCs

Multi-junction (three or more junction) APTSCs have the potential for even higher PCEs beyond 33%. Among the main works published thus far, the reported efficiencies, although lower than the double-junction tandem solar cell, were: 6.7% (Glass/FTO/SnO_2_/PC_61_BM/wide bandgap perovskite (WB PVK)/Spiro-MeOTAD/PEDOT:PSS/ITO NPs/PC_61_BM/intermediate bandgap perovskite (IB PVK)/Spiro-MeOTAD/PEDOT:PSS/ITO NPs/PC_61_BM/narrow bandgap perovskite (NB PVK)/Sprio-(TFSI)_2_/Ag), 16.8% (Glass/ITO/PTAA/WB PVK/C_60_/SnO_2_/Au/PEDOT:PSS/PTAA/IB PVK/C_60_/SnO_2_/Au/PEDOT:PSS/NB PVK/C_60_/BCP/Ag), 20.1% (Glass/ITO/PTAA/WB PVK/C_60_/ALD-SnO_2_/Au/NiO/PTAA/IB PVK/C_60_/ALD-SnO_2_/Au/PEDOT:PSS/NB PVK/C_60_/BCP/Cu), 24.3% (Glass/ITO/NiO_*x*_/Me-4PACz/WB PVK/PEAI-EDAI_2_/PCBM/PEIE/SnO_*x*_/ITO/NiO_*x*_/Me-4PACz/IB PVK/PEAI-EDAI_2_/PCBM/PEIE/SnO_*x*_/Au/PEDOT:PSS/NB PVK/C_60_/SnO_*x*_/Ag) and 25.1% (Glass/IO:H/NiO_*x*_/Me-4PACz/WB PVK/PCBM/PEI/ALD-SnO_*x*_/ITO/NiO_*x*_/Me-4PACz/IB PVK/C_60_/ALD-SnO_*x*_/Au/PEDOT:PSS/NB PVK/C_60_/ALD-SnO_*x*_/Ag).

Selecting the appropriate bandgap will be particularly important for the triple junction APTSCs as three different absorbers are needed (wide/intermediate/narrow bandgap). For example, publications demonstrated triple junction all-perovskite solar cells with bandgaps, as shown in Table 1. (Fig. 7d–f). Furthermore, the best stability of triple junction all-perovskite solar cells is currently 420 h, retaining 80% of their initial efficiency under illuminating and room temperature conditions from the literature. Hence, this triple junction field has promising potential for development of highly efficient and stable devices.^33–37^

**Table 1:** The bandgap data of triple junction perovskite solar cells

PCE (%)	Wide bandgap (eV)	Intermediate bandgap (eV)	Narrow bandgap (eV)
6.7^35^	1.94	1.57	1.34
16.8^37^	1.99	1.6	1.22
20.1^36^	1.73	1.57	1.23
24.3^33^	2	1.6	1.22
25.1^34^	1.97	1.61	1.25

**Table 2:** The chronological database of APTSCs

Device structure	Junction	Method	WB^b^ PCE (%)	WB (eV)	IB^c^ PCE (%)	IB (eV)	NB^d^ PCE (%)	NB (eV)	Tandem PCE (%)	Active area (mm^2^)	Terminal	Substrate	Accepted date (Ref.)
Glass/FTO/TiO_2_/WB PVK^a^/PTAA/PCBM/NB PVK/PEDOT:PSS/ITO	Double	Li-TFSI + *t*BP in HTM (P3HT or PTAA)	8.4	2.25	—	—	18	1.55	10.8	9.6	2T	Glass	Oct 2015^172^
Glass/FTO/c-TiO_2_/m-TiO_2_/WB PVK/Spiro-MeOTAD/PEDOT:PSS/PEI/PCBM:PEI/NB PVK/transparent hc-PEDOT:PSS	Double	Novel CRL	11.7	N/A	—	—	11.4	N/A	7	4–10	2T	Glass	Dec 2015^174^
WB: glass/ITO/NiO_*x*_/WB PVK/PCBM/bis-C_60_/ITO	Double	Solvent washing and compositional engineering	14.19	1.57^h^	—	—	14.35	1.33	19.08	3.14 or 10	4T	Glass	Aug 2016^143^
NB: glass/ITO/PEDOT:PSS/NB PVK/PCBM/bis-C_60_/Ag													
Glass/ITO/NiO/WB PVK/ZTO/SnO_2_/PCBM/PEDOT:PSS/NB PVK/C_60_/BCP/Ag	Double	Bandgap optimisation	9.8	1.8	—	—	14.1	1.2	16.9	20	2T	Glass	Oct 2016^153^
									20.1	20	4T		
									13.3	100	2T		
									16	100	4T		
Glass/ITO/TiO_2_/IPH/WB PVK/TaTm/TaTm:F6-TCNNQ/C_60_:Phlm/C_60_/NB PVK/TaTm/TaTm:F6-TCNNQ/Au	Double	Doped organic semiconductors	10.7	2	—	—	19.4	1.55	15.6	6	2T	Glass	Dec 2016^176^
WB: glass/FTO/SnO_2_/C_60_-SAM/WB PVK/Spiro-MeOTAD/MoO_*x*_/Au/MoO_*x*_	Double	Optimisation of PVK thickness	18.3	1.58	—	—	17.6	1.25	21.2	8.5	4T	Glass	Jan 2017^149^
NB: glass/ITO/PEDOT:PSS/NB PVK/C_60_/BCP/Ag							17.01^f^						
Glass/ITO/NiO_2_/WB PVK/C_60_/Bis-C_60_/Sputtered ITO/PEDOT:PSS/NB PVK/IC_60_BA/Bis-C_60_/Ag	Double	Indene-C_60_ bis-adduct (IC_60_BA) interface layer	12.5	1.82	—	—	14.4	1.22	18.4	10	2T	Glass	Jul 2017^181^
Glass/FTO/c-TiO_2_/m-TiO_2_/WB PVK/Spiro-MeOTAD/PEDOT:PSS/C_60_/NB PVK/Spiro-MeOTAD/Au	Double	Solution-processed organic charge recombination layer	5.2	2.3	—	—	12.7	1.55	5.1	N/A	2T	Glass	Dec 2017^175^
WB: glass/FTO/SnO_2_/C_60_-SAM/WB PVK/Spiro-MeOTAD/MoO_*x*_/ITO	Double	MoO_*x*_/ITO transparent electrodes	15.7	1.75	—	—	17.5	1.25	23.1	10	4T	Glass	Jan 2018^179^
NB: glass/ITO/PEDOT:PSS/NB PVK/C_60_/BCP/Ag													
ITO/PTAA/WB PVK/C_60_/SnO_2_/ITO/PEDOT:PSS/NB PVK/C_60_/BCP/Ag	Double	Compositional engineering using Cs^+^ replacing MA^+^	14.6	1.76	—	—	13.1	1.14	19.1	N/A	2T	Glass	Aug 2018^144^
Glass/ITO/PTAA/WB PVK/C_60_/BCP/Ag/MoO_*x*_/ITO/PEDOT:PSS/NB PVK/C_60_/BCP/Ag	Double	Incorporation of Cl^−^ for NB PVK	14	1.75	—	—	18.1	1.25	21	10.5	2T	Glass	Oct 2018^146^
Glass/ITO/TiO_*x*_/PC_61_BM/WB PVK/ICL (PTAA/HMB-Doped PC_61_BM)/NB PVK/HTL/Ag/Encapsulation	Double	Solution-processed interconnecting layer (ICL), HTL doping	18.29	1.58	—	—	15.55	1.26	18.69	12	2T	Glass	Oct 2018^173^
									16.2	120	2T		
ITO/NiO_*x*_/WB PVK/FSIP/C_60_/BCP/Cu/Au/PEDOT:PSS/NB PVK/polystyrene/C_60_/BCP/Ag	Double	Thermionic emission-based interconnecting layer (PSIP)	12.32	1.83	—	—	13.61	1.24	17.9	10	2T	Glass	Nov 2018^177^
Glass/FTO/SnO_2_/PC_61_BM/WB PVK/Spiro-MeOTAD/PEDOT:PSS/ITO NPs/PC_61_BM/IB PVK/Spiro-MeOTAD/PEDOT:PSS/ITO NPs/PC_61_BM/NB PVK/Spiro-(TFSI)_2_/Ag	Triple	Fully solution processed triple junction tandem	11.6	1.94	N/A	1.57	11.1	1.34	6.7	9.19	2T	Glass	Jan 2019^35^
WB: glass/ITO/PTAA/WB PVK/C_60_/BCP/Ag	Double	Guanidinium thiocyanate additive in NB PVK	18.5	1.63	—	—	20.5	1.25	23.1	10.5	2T	Glass	Apr 2019^161^
NB: glass/ITO/PEDOT:PSS/NB PVK/C_60_/BCP/Ag									25.4	10.5	4T	Glass	
PEN/ITO or glass/ITO/polytpd/PFN-Br/WB PVK/LiF/C_60_/PEIE/Aluminium-doped zinc oxide/ITO/PEDOT:PSS/NB PVK/C_60_/BCP/Au	Double	Recombination layer modification and cation tuning	19.2	1.7	—	—	16.5	1.27	23.1	5.8	2T	Glass	May 2019^62^
									21.3	5.8	2T	PEN	
Glass/ITO/PTAA/WB PVK/C_60_/BCP/ICL (Ag/MoO_3_/ITO)/PEDOT:PSS/NB PVK/C_60_/BCP/Ag	Double	Chlorine incorporation bulk-passivation	N/A	1.75	—	—	N/A	1.25	21.1	12	2T	Glass	Jun 2019^145^
Glass/ITO/PTAA/WB PVK/C_60_/ALD-SnO_2_/Au/PEDOT:PSS/NB PVK/C_60_/BCP/Cu	Double	Comproportionation reaction	16.5	1.77	—	—	21.1	1.22	24.8^f^	7.3	2T	Glass	Aug 2019^157^
									22.1^f^	105	2T		
									22.3	105	2T		
Glass/ITO/poly-TPD/PFN-Br/WB PVK/LiF/C_60_/PEIE/Aluminium-doped zinc/NB PVK/C_60_/BCP/Ag	Double	Band alignment of ITO-PVK heterojunction	16.6	N/A	—	—	15.4	1.29	20.8	5.8	2T	Glass	Aug 2019^120^
Glass/ITO/PTAA/WB PVK/C_60_/SnO_2_/Sputtered ITO/PEDOT:PSS/PTAA/NB PVK/C_60_/BCP/Cu	Double	Cadmium ion additive in NB PVK	16.3	1.8	—	—	20.3	1.22	23	6.84	2T	Glass	Sep 2019^158^
WB: glass/ITO/PTAA/WB PVK/C_60_/BCP/Ag	Double	Diluted PEDOT:PSS as a HTL	19.5	1.6	—	—	19.58	1.2	23.26	6	4T	Glass	Nov 2019^168^
NB: glass/ITO/PEDOT:PSS/NB PVK/C_60_/BCP/Ag													
WB: glass/ITO/SnO_2_/WB PVK/Spiro-MeOTAD/MoO_3_/ITO/MgF_2_	Double	Vacuum-assisted nucleation growth	18.5	1.63	—	—	18.2	1.27	23	100	4T	Glass	Dec 2019^147^
NB: glass/ITO/PEDOT:PSS/NB PVK/PCBM/C_60_/BCP/Ag													
Glass/ITO/PTAA/WB PVK/C_60_/ALD-SnO_2_/Au/PEDOT:PSS/NB PVK/C_60_/BCP/Cu	Double	Ultra-thin 2D layer using phenethylammonium cation for NB PVK	16.1	1.75	—	—	19.4	1.25	23.7	4.9	2T	Glass	Feb 2020^165^
							18.95^f^						
Glass/ITO/PTAA/WB PVK/(n^+^) C_60_/(n) SnO_2−*x*_/NB PKV/C_60_/BCP/Cu	Double	Simplified tandem structure	21.5	1.78	—	—	20.2	1.21	24.4	5.9	2T	Glass	Jun 2020^178^
									22.2	115			
Glass/ITO/PTAA/WB PVK/C_60_/ALD-SnO_2_/Au/NiO/PTAA/IB PVK/C_60_/ALD-SnO_2_/Au/PEDOT:PSS/NB PVK/C_60_/BCP/Cu	Triple	Triple multi-junction	10.4	1.99	19	1.6	20.1	1.22	20.1	4.9	2T	Glass	Aug 2020^36^
Glass/ITO/NiO/VNPB/WB PVK/C_60_/ALD-SnO_2_/Au/PEDOT:PSS/NB PVK/C_60_/ALD-SnO_2_/Cu	Double	Surface-anchoring zwitterionic molecules with formamidine sulfinic acid (FSA)	15.9	1.77	—	—	21.7	1.22	25.6	4.9	2T	Glass	Sep 2020^53^
									24.2^f^	100	2T		
							20.7^f^		21.4	1200	2T		
Triple junction: Glass/ITO/PTAA/WB PVK/C_60_/SnO_2_/Au/PEDOT:PSS/PTAA/IB PVK/C_60_/SnO_2_/Au/PEDOT:PSS/NB PVK/C_60_/BCP/Ag	Triple	Versatile two-step solution process	8.1	1.73	16.5	1.57	14.6	1.23	16.8	6.76	2T	Glass	Sep 2020^37^
Double junction: Glass/ITO/PTAA/WB PVK/C_60_/SnO_2_/Au/PEDOT:PSS/NB PVK/C_60_/BCP/Ag	Double								19.2				
WB: glass/ITO/np-SnO_2_/WB PVK/Spiro-MeOTAD/MoO_*x*_/ITO/MgF_2_	Double	Triple cation	18	1.65	—	—	18.2	1.26	23.6	10.5	4T	Glass	Oct 2020^154^
NB: glass/ITO/PTAA/NB PVK/PCBM/C_60_/BCP/Ag													
Glass/ITO/VNPB/WB PVK/C_60_/ALD-SnO_2_/Au/PEDOT:PSS/NB PVK/C_60_/BCP/Cu	Double	*N*_4_,*N*_4_'-di(naphthalen-1-yl)-*N*_4_,*N*_4_'-bis(4-vinylphenyl)biphenyl-4,4'-diamine (VNPB) as a HTL	16.7	1.77	—	—	15.9	1.16	24.9	4.9	2T	Glass	Jun 2021^169^
WB: glass/IO:H front electrode/np-SnO_2_/WB PVK/Spiro-MeOTAD/MoO_*x*_/IZO rear electrode/MgF_2_	Double	Hydrogen-doped indium oxide In_2_O_3_:H (IO:H) front electrode	19	1.62	—	—	17.3	1.26	24.8	N/A	4T	Glass	Sep 2021^180^
NB: glass/IO:H front electrode/NB PVK/PCBM/C_60_/BCP/Ag													
Glass/ITO/W-NiO_*x*_/WB PVK/C_60_/ALD-SnO_2_/ITO NCs/E-NiO_*x*_/NB PVK/C_60_/ALD-SnO_2_/Cu	Double	Metal oxides for HTLs and ETLs. E-NiO_*x*_ (nanocrystals NiO_*x*_ in ethanol) and W-NiO_*x*_ (nanocrystals NiO_*x*_ in water)	16.5	1.8^h^	—	—	17.4	1.24^h^	23.5	4.9	2T	Glass	Oct 2021^171^
WB: glass/IO:H/SnO_2_/WB PVK/Spiro-MeOTAD/MoO_*x*_/IZO/MgF_2_	Double	Interfacial engineering using indene-C_60_-propionic acid hexyl ester (IPH)	19	1.63	—	—	18.6	1.26	24.8	10.5	4T	Glass	Dec 2021^155^
NB: glass/ITO/PTAA/NB PVK/IPH/C_60_/BCP/Ag													
Glass/ITO/NiO/VNPB/WB PVK/C_60_/ALD-SnO_2_/Au/PEDOT:PSS/NB PVK/C_60_/BCP/Cu	Double	Passivation using 4-trifluoromethyl-phenylammonium (CF3-PA)	17.3	1.76	—	—	22.2	1.22^h^	26.7	4.9	2T	Glass	Dec 2021^163^
									26.4^f^				
Glass/ITO/NiO/VNPB/WB PVK/C_60_/ALD-SnO_2_/Au/PEDOT:PSS/NB PVK/C_60_/ALD-SnO_2_/Ag	Double	Blade coating and Cs additive	17.2	1.8	—	—	19	1.22^h^	25.1	4.9	2T	Glass	Apr 2022^56^
									22.5	2025^g^	2T		
									21.7^f^	2025^g^	2T		
Glass/ITO/PTAA/WB PVK/C_60_/ALD-SnO_2_/Au/PEDOT:PSS/NB PVK/C_60_/BCP/Cu	Double	Steric engineering using alloying dimethylammonium and chloride	17.7	1.8	—	—	20.7	1.2	26.2	N/A	2T	Glass	Apr 2022^152^
Glass/MeO-2PACz/WB PVK/LiF/C_60_/SnO_*x*_/Au/PEDOT:PSS/NB PVK/C_60_/BCP/Ag	Double	Quasi-2D with bulky organic cations phenethylammonium and guanidinium	16.6	1.75	—	—	22.2	1.25	25.5	9	2T	Glass	May 2022^103^
									24.3^f^				
PEN/ITO/MB-NiO/WB PVK/C_60_/ALD-SnO_2_/Au/PEDOT:PSS/NB PVK/C_60_/BCP/Cu	Double	Mixture of two hole-selective molecules (MB-NiO referred to a molecule-bridged NiO)	16.2	1.75	—	—	N/A	1.22	24.7	4.9	2T	Flexible	May 2022^63^
									24.4^f^	4.9			
									23.5	105			
MgF_2_/glass/IO:H/2PACz/WB PVK/LiF/C_60_/SnO_*x*_/ITO or Au/PEDOT:PSS/NB PVK/PCBM/C_60_/BCP/Cu	Double	Up-scaling with blade coating and vacuum deposition	N/A	1.78	—	—	N/A	1.26^h^	23.5	10	2T	Glass	May 2022^55^
									19.1	1225^g^	2T		
									19.8	243^g^	2T		
									18.3^f^	243^g^	2T		
WB: PET/ITO/SnO_2_/WB PVK/Spiro-MeOTAD/VO_*x*_/IMI	Double	Guanidinium thiocyanate additives in PVK	9.52	1.71	—	—	9.27	1.26	15.3	5000^g^	4T	Flexible	Jun 2022^54^
NB: PET/ITO/PEDOT:PSS/NB PVK/C_60_/BCP/Cu													
Glass/ITO/PTAA/WB PVK/C_60_/SnO_2_/ITO/PEDOT:PSS/NB PVK/C_60_/BCP/Ag	Double	Close-space annealing	18.58	1.57	—	—	21.51	1.25	25.15	9	4T	Glass	Jun 2022^148^
									25.05	9	2T		
									24.79^f^	9	2T		
Glass/ITO/SnO_2_/(2D/3D)WB PVK/Spiro-MeOTAD/PEDOT:PSS/C_60_/NB PVK/Spiro-OMeTAD/Ag	Double	*In situ* growth using *n*-butylammonium bromide	16.07	1.57	—	—	15.07	1.59	10.22	4	2T	Glass	Jun 2022^164^
Glass/ITO/PTAA/WB PVK/C_60_/SnO_2_/Au/PEDOT:PSS/NB PVK/PCBM/C_60_/BCP/Cu	Double	Hot gas-assisted blade-coating	16.4	1.67	—	—	20.3	1.22	23.1	8	2T	Glass	Jul 2022^57^
									21.6	1430^g^	2T		
WB: PEN/ITO/2PACz/PTAA/WB PVK/TEACl/PCBM/np-ZnO/IZO	Double	Post-treatment using 2-thiopheneethylammonium chloride (TEACl)	15.1	1.77	—	—	18.2	1.24	22.6	9	4T	Flexible	Sep 2022^61^
NB: PEN/ITO/PEDOT:PSS/NB PVK/C_60_/BCP/Cu									23.8	9	2T		
2T: PEN/ITO/2PACz/PTAA/WB PVK/TEACl/PCBM/ALD-SnO_2_/ITO/PEDOT:PSS/NB PVK/C_60_/BCP/Cu													
Glass/ITO/NiO/SAM/WB PVK/C_60_/SnO_2_/Au/PEDOT:PSS/NB PVK/C_60_/SnO_2_/ITO	Double	Bifacial tandem	N/A	1.68	—	—	N/A	1.22	28.51 (bifacial with RI^e^)	9	2T	Glass	Sep 2022^128^
Glass/ITO/MeO-2PACz/WB PVK/C_60_/Au/SnO_*x*_/PEDOT:PSS/NB PVK/C_60_/BCP/Cu	Double	Dual interface treatment using MeO-2PACz self-assembled monolayer (SAM) and ethylenediammonium diiodide	17.8	1.76	—	—	19.6	1.28	24.1	20	2T	Glass	Sep 2022^182^
WB: Glass/ITO/SnO_2_/WB PVK/Spiro-MeOTAD/MoO_*x*_/ITO NB: Glass/ITO/PEDOT:PSS/NB PVK/PCBM/BCP/Ag	Double	Tuning bandgap and fully inorganic PVK film of WB and NB	11.26	1.98	—	—	13.8	1.39	18.07	10	4T	Glass	Oct. 2022^232^
Glass/ITO/MeO-2PACs/WB PVK/C_60_/ALD-SnO_2_/graphene oxide/PEDOT/resin/NB PVK/C_60_/ALD-SnO_2_/ITO	Double	Bifacial tandem	N/A	1.65	—	—	19.4	1.21	24.4 (mono)	8	2T	Glass	Oct 2022^130^
									29.3 (bifacial with RI)				
Glass/ITO/NiO/VNPB/WB PVK/C_60_/ALD-SnO_2_/ITO NCs/NB PVK/C_60_/ALD-SnO_2_/Cu	Double	Fully FA A-site ion based perovskite film	17.3	1.77	—	—	21	1.26	26.3	4.9	2T	Glass	Oct 2022^121^
Glass/FTO/PEDOT:PSS/NB PVK/C_60_/n-C_60_/p-TaTm/TaTm/WB PVK/C_60_/BCP/ITO PLD/Ag/ALD Al_2_O_3_	Double	Vacuum-deposited organic charge recombination layer	12.7	1.68	—	—	17.5	1.26	20.1	N/A	2T	Glass	Nov 2022^129^
Glass/ITO/NiO_*x*_/Me-4PACz/WB PVK/C_60_/ALD-SnO_*x*_/Au/PEDOT:PSS/NB PVK/C_60_/ALD-SnO_*x*_/Ag	Double	Passivation using 1,3-propane diammonium	20.2	1.79	—	—	21.5	1.22	27.4	4.9	2T	Glass	Nov 2022^156^
			19.3^f^						26.29^f^	4.9			
Glass/ITO/SAM/CuBr/WB PVK/CuBr/LiF/C_60_/ALD-SnO_2_/Au/PEDOT/NB PVK/C_60_/BCP/Cu	Double	PSS-free PEDOT	20.5	1.75	—	—	20.1	1.25	21.5	7.06	2T	Glass	Nov 2022^167^
WB: PEN/ITO/2PACz/WB PVK/PCBM/ZnO/IZO	Double	SnF_2_ additive	15.2	1.78	—	—	18.5	1.24	23.1	9	4T	Flexible	Jan 2023^60^
NB: PEN/ITO/PEDOT:PSS/NB PVK/C_60_/BCP/Cu													
N/A	Double	Tris(2,4-pentanedionato)gallium additive	17.03	1.74	—	—	20.76	1.25	23.14	8	4T	Glass	Mar 2023^162^
							6.11 (filtered)						
Glass/ITO/2PACz/WB PVK/C_60_/SnO_2_/ITO/PEDOT:PSS/CysHCl-D&P NB PVK/C_60_/BCP/Cu	Double	Passivation using cysteine hydrochloride (CysHCl)	17.48	1.77	—	—	22.15	1.27	25.7^f^	5.76	2T	Glass	Mar 2023^26^
Glass/ITO/PTAA/PFMBr/WB PVK/C_60_/SnO_2_/ITO/PEDOT:PSS/NB PVK/C_60_/BCP/Cu	Double	PbCl_2_ and phenethylammonium chloride (PMACl) additive	20.22	1.73	—	—	21.97	1.25	26.68	9	2T	Glass	Mar 2023^28^
Glass/ITO/NiO/SAM/WB PVK/C_60_/ALD-SnO_2_/Au/PEDOT:PSS/NB PVK/C_60_/BCP/Cu	Double	Passivation using 4-(Trifluoromethyl) phenethylammonium	18.5	1.78	—	—	21.7	1.22	25.6	4.9	2T	Glass	Mar 2023^131^
WB: ITO/MeO-2PACz/WB PVK/C_60_/ALD-SnO_2_/ITO	Double	Antimony potassium tartrate additive	20.35	1.67	—	—	20.8	1.24^h^	26.3	11.88	4T	Glass	Mar 2023^29^
NB: ITO/PEDOT:PSS/NB PVK/C_60_/BCP/Cu							7.6 (filtered)						
Glass/Cu/ITO/PEDOT:PSS/NB PVK/C_60_/ALD-SnO_2_/ITO/NiO/SAM/C_60_/ALD-SnO_2_/IZO/Anti-reflection layer	Double	GuaBF_4_ additive	19.1	1.77	—	—	19.6	1.22	25.3	5.29	2T	Glass	Mar 2023^132^
									24.1	5.29	2T	Flexible (Cu-PEN)	
									20.3	5.29	2T	Flexible (Cu foil)	
Glass/ITO/novel HTL/WB PVK/C_60_/SnO_2_/IZO/NB PVK/C_60_/SnO_2_/Cu	Double	hole-selective layer using self-assembled monolayer of (4-(7*H*-dibenzo[*c,g*]carbazol-7-yl)butyl)phosphonic acid	18.22	1.77	—	—	21.27	1.25	27	104.4	2T	Glass	Mar 2023^31^
									26.4^f^				
Glass/ITO/NiO_*x*_/Me-4PACz/WB PVK/PEAI-EDAI_2_/PCBM/PEIE/SnO_*x*_/ITO/NiO_*x*_/Me-4PACz/IB PVK/PEAI-EDAI_2_/PCBM/PEIE/SnO_*x*_/Au/PEDOT:PSS/NB PVK/C_60_/SnO_*x*_/Ag	Triple	Rb/Cs mixed cation inorganic perovskite	13.4	2	N/A	1.6	N/A	1.22	24.3	4.9	2T	Glass	Mar 2023^34^
									23.2^f^				
Glass/ITO/PTAA/4,3BuPACz/WB PVK/C_60_/ALD-SnO_2_/IZO/PEDOT:PSS/NB PVK/C_60_/BCP/Ag	Double	PTAA/monomolecular substrate	16.57	1.77	—	—	N/A	1.25^h^	25.24	10	2T	Glass	Mar 2023^170^
Glass/ITO/2F/WB PVK/C_60_/ALD-SnO_2_/IZO/PEDOT:PSS/2F/NB PVK/C_60_/ALD-SnO_2_/Cu	Double	hole selective contact using 4-(7-(4-(bis(4-methoxyphenyl)amino)-2,5-difluorophenyl)benzo[c][1,2,5]thiadiazol-4-yl) benzoic acid (2F)	19.33	1.77	—	—	23.24	1.25	27.22	5.76	2T	Glass	May 2023^30^
			19.09^f^						26.3^f^				
Glass/ITO/2PACz/WB PVK/C_60_/SnO_*x*_/Au/PEDOT:PSS/NB PVK/C_60_/BCP/Cu	Double	Natural SnO_*x*_ doping	N/A	1.78	—	—	22.16	1.25	26.01	9.48	2T	Glass	May 2023^32^
Glass/ITO/NiO/SAM/WB PVK/C_60_/ALD-SnO_2_/Au/PEDOT:PSS/NB PVK/full-lead WB/C_60_/BCP or ALD-SnO_2_/Cu	Double	3D/3D bilayer	18.6	1.78	—	—	23.8	1.25	28.5	4.9	2T	Glass	May 2023^5^
									28^f^				
Glass/ITO/2PACz-SAM/WB PVK/LiF/C_60_/SnO_2_/Au/PEDOT:PSS/NB PVK/C_60_/PEIE/Ag	Double	Carbazole moiety-based ([2-(9*H*-carbazol-9-yl)ethyl]phosphonic acid) self-assembled monolayer treatment (2PACz-SAM) for WB PVK	17.62	1.77	—	—	18	1.24	24.66	9.3	2T	Glass	Sep 2023^183^
			16.72^f^										
Glass/IO:H/NiO_*x*_/Me-4PACz/WB PVK/PCBM/PEI/ALD-SnO_*x*_/ITO/NiO_*x*_/Me-4PACz/IB PVK/C_60_/ALD-SnO_*x*_/Au/PEDOT:PSS/NB PVK/C_60_/ALD-SnO_*x*_/Ag	Triple	Diammonium halide salt and propane-1,3-diammonium iodide	15.5	1.97	21.3	1.36	20.1	1.25	25.1	4.9	2T	Glass	Oct 2023^33^
									23.8^f^				
Glass/ITO/NiO_*x*_/Me-4PACz/WB PVK/C_60_/ALD-SnO_2_/Au/PEDOT:PSS/NB PVK/C_60_/ALD-SnO_2_/Ag	Double	Potassium hypophosphite additive into the wide bandgap perovskite solution	20.06	1.79	—	—	21.73	1.25	26.08	7.8	2T	Glass	Nov 2023^184^
WB: glass/ITO/DCB-BPA/WB PVK/C_60_/SnO_2_/IZO	Double	Self-assembled monolayer of (4-(5,9-dibromo-7*H*-dibenzo[*c*,*g*]carbazo-7-yl)butyl)phosphonic acid (DCB-BPA) as the hole-selective layer	18.88^f^	1.77	—	—	20.53	1.25	26.9	9.75	4T	Glass	Nov 2023^185^
NB: glass/PEDOT:PSS/NB PVK/C_60_/BCP/Cu													
WB: glass/ITO/MeO-2PACz/WB PVK/C_60_/SnO_2_/ITO	Double	Dodecyl-benzene-sulfonic acid additive in wide bandgap perovskite precursor solution	22.4	1.66	—	—	21.24	1.25	28.06	7.022	4T	Glass	Nov 2023^186^
			21.97^f^										
NB: glass/ITO/PEDOT:PSS/NB PVK/C_60_/BCP/Cu			20.13										
Glass/ITO/(4-(7*H*-dibenzo[*c*,*g*]carbazole-7-yl)butyl) phosphonic acid/WB PVK/C_60_/ALD-SnO_2_/ITO/Taurine/NB PVK/C_60_/BCP/Cu	Double	HTL free NB PVK using 2-aminoethanesulfonic acid	N/A	1.72^h^	—	—	22.5	1.25	26.03	9.75	2T	Glass	Nov 2023^187^
Glass/ITO/NiO_*x*_/SAMs/WB PVK/HF-WB PVK/ALD-SnO_2_/Au/PEDOT:PSS/NB PVK/HF-NB PVK/ALD-SnO_2_/CDB/Cu	Double	Hybrid fullerenes comprising a mixture of fullerene, phenyl C_61_ butyric acid methylester, and indene-C_60_ bisadduct	19	1.8	—	—	20.6	1.25	23.3	2025^g^	4T	Glass	Nov 2023^188^
Glass/FTO/NiO_2_/MeO-2PACz/LiF/C_60_/ALD-SnO_2_/Au/IE-PEDOT:PSS/NB PVK/C_60_/PEIE/Ag	Double	Doping MAI-DMSO in PEDOT:PSS	N/A	1.77	—	—	21.28	1.25	23.52	17.64	2T	Glass	Nov 2023^189^
Anti-reflection foil/glass/ITO/2PACz/WB PVK/C_60_/SnO_2_/ITO/PEDOT:PSS/NB PVK/C_60_/PEIE/SnO_2_/Cu	Double	Piperazinium iodide surface treatment for wide bandgap perovskite and rubidium iodide additive for narrow bandgap perovskites	20.3	1.8	—	—	20.6	1.26	27.5^f^	12	2T	Glass	Nov 2023^190^
WB: glass/ITO/PTAA/WB PVK/C_60_/ZnO	Double	Triethanolamine borate additive in wide bandgap precursor solution	21.55	1.65	—	—	21.41	1.25	26.48	N/A	4T	Glass	Nov 2023^191^
NB: glass/ITO/PEDOT:PSS/NB PVK/C_60_/BCP/Ag							7.32 (filtered)						
Glass/ITO/NiO_*x*_/Me-4PACz/WB PVK/C_60_/ALD-SnO_2_/ITO/PEDOT:PSS/NB PVK/C_60_/BCP/Ag	Double	Surface reaction layer with ethanediamine dihydroiodide	23.1	1.67	—	—	22.1	1.25	26.1	7	2T	Glass	Dec 2023^192^
			22.95^f^	1.67									
			19.7	1.75									
			18.81^f^	1.75									
Glass/ITO/NiO_*x*_/MeO-2PACz/WB PVK/C_60_/SnO_*x*_/Au/PEDOT:PSS/NB PVK/C_60_/BCP/Ag	Double	Chloromethylphosphonic acid additive in tin–lead perovskites	19.6	1.79	—	—	21.6	1.25	27.3	4.2	2T	Glass	Dec 2023^193^
									26.96^f^				
Glass/ITO/NiO/VNPB/Me-4PACz/WB PVK/C_60_/ALD-SnO_2_/Au/PEDOT:PSS/NB PVK/C_60_/ALD-SnO_2_/Cu	Double	Aminoacetamide hydrochloride additive in Sn–Pb perovskite film	17.9	1.8	—	—	21.4	1.25	26.8	10	2T	Glass	Dec 2023^58^
									24.9	2000^g^			
									24.5^f^	2025^g^			
									23.8	6400^g^			
WB: glass/ITO/MeO-2PACz/WB PVK/C_60_/SnO_2_/ITO	Double	Blade coated perovskite and post-treatment with 1,3-propane-diammonium iodide and formamidinium iodide	18.71	1.77	—	—	9.3 (filtered)	1.25	27.64	102	4T	Glass	Jan 2024^194^
NB: glass/ITO/PEDOT:PSS/NB PVK/C_60_/BCP/Cu													
Substrate/ITO/NiO_*x*_/N719/WB PVK/C_60_/ALD-SnO_2_/Au/PEDOT:PSS/NB PVK/C_60_/BCP/Cu	Double	Methylene diphenyl diisocyanate polyurethane and PJ71 in wide and narrow bandgap perovskite solution	17.6	1.8	—	—	20.9	1.2	26.1	9	2T	Glass	Jan 2024^195^
									22.2	9	2T	PEN	
									11.6	9	2T	Silk-derived substrate	
Glass/ITO/NiO_*x*_/Me-4PACz/WB PVK/C_60_/ALD-SnO_*x*_/1nm Au/PEDOT:PSS/NB PVK/C_60_/ALD-SnO_*x*_/Ag	Double	Oleic acid treatment for Sn–Pb perovskite film	20	1.79	—	—	23	1.24	27.3	4.9	2T	Glass	Jan 2024^196^
									26.4^f^				
Glass/ITO/Me-4PACz/WB PVK/C_60_/SnO_2_/Au/PEDOT:PSS/NB PVK/C_60_/SnO_2_/Ag	Double	Tin(ii) oxalate additive in tin–lead perovskite solution	N/A	N/A	—	—	23.36	1.25	27.56	9	2T	Glass	Jan 2024^197^
Glass/ITO/Me-4PACz/WB PVK/SEBr/LiF/C_60_/ALD-SnO_2_/Au/PEDOT:PSS/NB PVK/C_60_/BCP/Ag	Double	Surface treatment using S-ethylisothiourea hydrobromide (SEBr) for WB PVK	22.47	1.67	—	—	N/A	1.25	27.1	5.8	2T	Glass	Jan 2024^198^
			19.9	1.77									
Glass/ITO/NiO_*x*_/2PACz/WB PVK/C_60_/SnO_2_/PEDOT:PSS/NB PVK/C_60_/BCP/Ag	Double	Surface treatment using the solution combined 3-fluorophenethylammonium iodide and ethane-1,2-diammonium iodide	19.31^f^	1.77	—	—	21.39	1.25	27.04^f^	4.2	2T	Glass	Feb 2024^199^
Glass/ITO/PTAA/WB PVK/C_60_/ALD-SnO_2_/Au/PEDOT:PSS/NB PVK/C_60_/BCP/Ag	Double	Benzyl viologen post-treatment of narrow bandgap perovskites	17.83	1.78	—	—	22.58	1.25	26.33	6	2T	Glass	Feb 2024^200^
WB: Glass/ITO/NiO_*x*_/Me-4PACz/WB PVK/C_60_/SnO_2_/ITO	Double	Octane-1,8-diamine dihydroiodide additive in wide bandgap perovskite solution	20.06	1.67	—	—	22.75	1.25	28.35	100	4T	Glass	Mar 2024^201^
NB: glass/ITO/PEDOT:PSS/NB PVK/C_60_/BCP/Cu							8.29 (filtered)						
Glass/ITO/NiO_*x*_/2PACz/WB PVK/C_60_/ALD-SnO_2_/ITO nano crystals/PEDOT:PSS/NB PVK/C_60_/SnO_2_/Cu	Double	2D passivation using 2-(4-fluorophenyl)ethylamine hydroiodide on WB PVK	19.4	1.78	—	—	21.5	1.22	27	6.84	2T	Glass	Apr 2024^202^
WB: glass/ITO/PTAA/WB PVK/C_60_/PEI/ITO	Double	Polyethylenimine (PEI) for high energy sputtered ITO	19.17	1.66	—	—	22.1	1.24	26.89	4	4T	Glass	Apr 2024^203^
NB: glass/ITO/PEDOT:PSS/NB PVK/C_60_/BCP/Ag			18.09^f^				7.72 (filtered)						
WB: glass/ITO/MeO-2PACz/WB PVK/PDAI_2_/C_60_/ALD-SnO_2_/ITO/Ag	Double	Surface passivation using 1,3-propane-diammonium iodide (PDAI_2_) for WB PVK	20.11	1.68	—	—	21.26	1.21^h^	28.07	11.88	4T	Glass	Apr 2024^204^
NB: glass/ITO/PEDOT:PSS/NB PVK/C_60_/BCP/Cu							7.96 (filtered)						

a PVK: perovskite.

b WB: wide bandgap.

c IB: intermediate bandgap.

d NB: narrow bandgap.

e RI: rear irradiance.

f Certified device (marked in WB PCE (%), NB PCE (%) and tandem PCE (%)).

g Module (marked in the active area).

h Bandgap information (WB, IB or NB) was extracted manually from EQE or IPCE graphs.

**Table 3:** The database of simulated APTSCs

Device structure	Junction	Method	Ideal *V*_oc_ (V)	Ideal *J*_sc_ (mA cm^−2^)	Ideal FF (%)	Ideal PCE (%)	Type	Year (Ref.)
2-junction: MgF_2_/glass/ITO/NiO_*x*_/WB PVK 1.5–2 eV/C_60_/SnO_2_/ITO/PEDOT:PSS/NB PVK (0.9–1.35 eV)/C_60_/Ag	Double	Bandgap optimisation of PVK absorber layer	2.17	18.3	84	33.4	2T	2017^205^
3-junction: MgF_2_/glass/ITO/NiO_*x*_/WB PVK (1.9–2.3 eV)/C_60_/SnO_2_/ITO/NiO_*x*_/IB PVK (1.5–1.9 eV)/C_60_/SnO_2_/ITO/PEDOT:PSS/PVK (1.22 eV)/C_60_/Ag	Triple		3.54	12	86	36.6	2T	
NiO/WB PVK/PCBM/recombination layer/PEDOT:PSS/NB PVK/C_60_	Double	Bandgap and thickness optimisation of PVK absorber layer and study of contact energy levels, charge carrier mobility, and transport layer doping	N/A	N/A	N/A	36.6	2T	2019^206^
WB: NiO/Cs_2_AgBi_0.75_Sb_0.25_Br_6_	Double	Thickness optimisation of PVK absorber layer	1.83	14.9	63.57	17.35	4T	2020^209^
NB: PEDOT:PSS/FACsPb_0.55_Sn_0.5_I_3_/C_60_/BCP								
WB: Spiro-MeOTAD/Cs_2_AgBi_0.75_Sb_0.25_Br_6_/PCBM/ZnO	Double	Thickness optimisation of PVK absorber layer	1.94	15.55	68.48	24.86	4T	2020^210^
NB: Cu_2_O/CH_3_NH_3_SnI_3_/ZnO								
FTO/ZnO/CH_3_NH_3_GeI_3_/p^+^/n^+^/FAMASnGeI_3_/Cu_2_O/Au	Double	Thickness optimisation of PVK absorber layer and the study of influence of defect density on photovoltaic performance	1.07	28.36	84.46	26.72	2T	2020^211^
Glass/ITO/NiO_*x*_/WB PVK/SnO_2_/PCBM/ITO/PEDOT:PSS/NB PVK/C_60_/Ag	Double	Thickness optimisation of layer and optical optimisation considering materials	N/A	N/A	N/A	37	2T	2020^214^
2T: glass/FTO/PCBM/WB PVK CsSn_0.5_Ge_0.5_I_3_ (1.5 eV)/Spiro-MeOTAD/recombination layer/TiO_2_/NB PVK CsSnI_3_ (1.3 eV)/PTAA/Au	Double	Thickness optimisation of PVK absorber layer	WB: 0.85	21.22	71.18	18.32	2T	2021^212^
4T: WB: glass/PCBM/WB CsSn_0.5_Ge_0.5_I_3_ (1.5 eV)/Spiro-MeOTAD			NB: 0.35	WB: 25.49	WB: 73.36	Sum: 19.86	4T	
NB: TiO_2_/NB CsSnI_3_ (1.3 eV)/PTAA			Sum: 1.21	NB: 18.54	NB: 61.12			
TiO_2_/MAGeI_3_/Spiro-MeOTAD/ITO/FTO/TiO_2_/FASnI_3_/Spiro-MeOTAD	Double	Thickness optimisation of PVK absorber layer	2.63	14.6	80	30.85	2T	2021^213^
N/A	Double	Thickness optimisation of transparent electrode layer	N/A	N/A	N/A	24.4	2T	2021^215^
Glass/FTO/ZnOS/CsPbIBr_2_/GuAlO_2_/ITO/TiO_2_/MAPbI_3_/Spiro-MeOTAD/Au	Double	Thickness optimisation of PVK absorber layer and study of interface defects	2.6	12.21	86.42	27.4	2T	2021^207^
ITO/Cu_2_O/WB PVK FA_0.8_Cs_0.2_Pb(I_0.7_Br_0.3_)_3_/PCBM/SnO_2_/ITO/PEDOT:PSS/NB PVK (FASnI_3_)_0.6_(MAPbI_3_)_0.4_:Cl/PCBM/SnO_2_/Ag	Double	Thickness optimisation of various transport layers	2.05	18.3	86.23	32.3	2T	2022^216^
WB: ITO/TiO_2_/WB MAGeI_3_/Spiro-MeOTAD/ITO/MoO_*x*_	Double	Study regarding buffer layer and anti-reflection layer as well as nano texture pattern	WB: 1.7	WB: 8.63	WB: 92	WB: 13.5	4T	2022^217^
NB: ITO/TiO_2_/NB MASnI_3_/Spiro-MeOTAD/Ag			NB: 0.93	NB: 23.83	NB: 75	NB: 16.64		
PET (flexible) substrate						Sum: 30.1		
TiO_2_/WB PVK/Spiro-MeOTAD/C_60_ & IC_60_BA/NB PVK/PEDOT:PSS	Double	Optimisation of the transport layers and thickness of PVK absorber layers	1.95	15.21	74.09	21.97	2T	2022^218^
Glass/ITO/NiO/VNPB/WB PVK (1.77 eV)/C_60_/ALD-SnO_2_/Au 1 nm/PEDOT:PSS/NB (1.22 eV) PVK/C_60_/ALD-SnO_2_/Cu	Double	Thickness optimisation of PVK absorber layers and research regarding trap density and carrier mobilities	N/A	N/A	N/A	>30	2T	2022^219^
2T: Antirefelction coating (ARC)/ITO/NiO/WB PVK/SnO_2_/ITO/PEDOT:PSS/NB PVK/C_60_/Ag	Double	Researching all-inorganic based-APTSCs	WB: 1.37	WB: 18.05	WB: 84.64	WB: 20.91	4T	2023^220^
						NB: 9.54		
4T WB: Antireflection coating/ITO/NiO/WB PVK/SnO_2_/ITO			NB: 0.81	NB: 14.73	NB: 79.68	Sum: 30.5	2T	
NB: ITO/PEDOT:PSS/NB PVK/C_60_/Ag			Sum: 2.16	16	80.68	27.86		
Monofacial: ARC/glass/IO:H/WB PVK/C_60_/SnO_2_/ITO/PEDOT/NB PVK/PCBM/C_60_/Ag	Double	Monofacial (MF) and bifacial (BF) modelling study	2.25 (MF)	17.6 (MF)	81 (MF)	31.9 (MF)	2T (MF)	2023^221^
Bifacial: ARC/glass/IO:H/WB PVK/C_60_/SnO_2_/ITO/PEDOT/NB PVK/PCBM/C_60_/ITO/encapsulation/glass/ARC			2.25 (BF)	17.0 (BF)	81 (BF)	30.8 (BF)	2T (BF)	
ITO/SnO_2_/PFN-Br/WB PVK (1.75 eV)/Cu_2_O/ITO/SnO_2_/PCBM/NB PVK (1.25 eV)/PEDOT:PSS/Ag	Double	Beyond 2V in APTSCs	WB: 0.804	WB: 18	WB: 87.61	WB: 19.63	2T	2023^222^
			NB: 2.049	NB: 18	NB: 84.21	NB: 12.19		
			Sum: 2.05	Sum: 18	Sum: 86.3	Sum: 31.8		
LiF/PET/ITO/NiO_*x*_/WB PVK/C_60_/SnO_2_/ITO/PEDOT:PSS/NB PVK/C_60_/SnO_2_/Ag	Double	Flexible APTSCs	2.0328	18.38	93.21	34.83	2T	2023^223^
MgF_2_/FTO/TiO_2_/WB PVK (MAGeI_3_: 1.9 eV)/Spiro-MeOTAD/MoO_*x*_/IOH/TiO_2_/NB PVK (MASnI_3_: 1.3 eV)/Spiro-MeOTAD/Au	Double	Lead free perovskite and	2.71	14.06	87	33.14	2T	2024^224^
		thickness optimisation						
ITO/SnO_2_/C_60_/PCBM/WB PVK(1.99 eV)/Me-4PACz/IB PVK(1.6 eV)/C_60_/PCBM/ITO/Spiro-MeOTAD/NB PVK(1.2 eV)/PCBM/C_60_	Triple	Thickness optimisation	3.76	10.7	69	26.24	2T	2024^225^
WB: FTO/TiO_2_/WB PVK (MAGeI_3_: 1.9 eV)/Au	Double	HTL free, non-toxic perovskite and	1.3681	15.85	75.95	25.69	4T	2024^226^
NB: FTO/ZnO/NB PVK (FASnI_3_: 1.4 eV)/Au		thickness optimisation						
ITO/NiO_*x*_/WB PVK/ZnO/NB PVK (CuO/Cu/Al_2_O_3_)/SnO_2_/Ag	Double	Three-terminal	WB: 1.34	WB: 16.05	WB: 82.04	WB: 17.64	3T	2024^227^
			NB: 0.806	NB: 21.75	NB: 82.83	NB: 14.52		
			Sum: 2.146	Sum: N/A	Sum: N/A	Sum: 32.16		
WB: FTO/TiO_2_/WB PVK (CsGeI_3_: 1.6 eV)/Spiro-MeOTAD/Au	Double	Lead free perovskite and	2.12	16.71	85.87	30.42	4T	2024^228^
NB: FTO/ZnO/NB PVK (FASnI_3_: 1.41 eV)/Spiro-MeOTAD/Au		thickness optimisation						
WB: TiO_2_/WB PVK (MAPbI_3_)/Cu_2_O	Double	Thickness optimisation and	1.68	24.6	79.39	32.74	4T	2024^229^
NB: ZnO/NB PVK (MASnI_3_)/Cu_2_O		effect of defect density						

## 6. Challenges and solutions for APTSCs

PSCs have rapidly developed efficiency and stability. The latest research has demonstrated that APTSCs showed efficiencies higher than single-junction PSCs. However, in APTSCs, the stability-related degradation issue still remains a challenge, *i.e.*, the phase transition from the original crystal structure to the constituent components or a side phase caused by heat, freezing, moisture, or illumination. Herein, various methods were described on how APTSCs accelerated efficiency and stability. Furthermore, we detailed recent research trends in comprehensive summary graphs and tables (Fig. 1a–d, Table 2 and Table S1, ESI†).

### 6.1. Perovskite absorber layers

The perovskite absorber layer is among the most challenging to modify in the APTSC architecture, as it is the core layer directly correlated to efficiency and stability. For example, Sn-based perovskites generally used for achieving narrow bandgaps have poor moisture stability due to the oxidation of Sn^2+^ to Sn^4+^. Another example is that many defects exist on the perovskite surface area, which may cause non-radiative recombination. Additionally, wide bandgap perovskites have a *V*_oc_ loss challenge.

#### 6.1.1. Crystal growth control

Crystal growth control is crucial for the device performances. The crystal nuclei originate from particles such as organic molecules or ions in the perovskite precursor solution. These particles move randomly in a perovskite precursor solution, forming (statistically and at a low percentage) clusters, which typically disappear rapidly in an unsaturated solution system. If the perovskite precursor is oversaturated, the seed crystal from clusters can grow and exceed the critical seed size to trigger crystal growth.^133^

The LaMer mechanism and Ostwald ripening are well-established theories regarding crystal growth. The LaMer mechanism consists of three steps. The first step is to increase the concentration of the solution until a critical point. The next step shows rapid nucleation and crystal growth formation once the concentration is oversaturated, which means crossing a certain energy barrier. Meanwhile, the concentration will start reducing. If the concentration of the solution is sufficiently low, the nucleation starts reducing. In the final step, the diffusion in the solution dominates.^134^ As another crystal growth model, Ostwald ripening occurs when small particles dissolve into larger particles due to the high surface energy and high solubility of small particles.^135–137^ Especially, some perovskite studies applied a digestive ripening model theory, which is the inverse of Ostwald ripening, expecting the increase of bonds with ligands on the surface or prevention of organic component loss during thermal annealing.^138–141^

Generally, the small grain size is a potential challenge for efficiency and stability because grain boundaries are potentially the sites for defects, which can affect the performances through increased recombination and thus limit the open-circuit voltage.^142^ For these reasons enlarging the grain size is a strategy for improving the efficiency and stability of PSCs.

Dimethyl sulfoxide (DMSO) is often used as a co-solvent in the perovskite precursor due to its property of retarding crystallisation while forming intermediate complexes.^89^ In the 25% tin incorporated narrow bandgap perovskite precursor (MAPb_0.75_Sn_0.25_I_3_), the DMSO co-solvent created the intermediate phase, SnI_2_·3DMSO. This phase led to the production of high-quality perovskite films by slow crystallisation.^143^ In contrast, the volatile acetonitrile (CH_3_CN)/methylamine (CH_3_NH_2_) (ACN/MA) solvent-based perovskite solution process was demonstrated to have a faster drying speed than DMF.^35^

Methylammonium chloride (MACl) vapour surface treatment affected grain growth significantly to the micron size and can heal defects like cracks.^144^ Chloride (Cl^−^) is one of the favourable elements in the single junction perovskite composition, as it enhances the grain size. Cl^−^ has also improved the device performance of monolithic all-perovskite solar cells by increasing the grain size and reducing the electronic disorder in the Sn–Pb-based narrow bandgap perovskite layers.^145,146^

The vacuum-assisted growth control (VAGC) method showed reproducible film quality by enlarging the grain size from <400 nm to 2 μm in the narrow bandgap perovskite solar cell (FA_0.8_MA_0.2_Sn_0.5_Pb_0.5_I_3_). The primary purpose of the VAGC method was to form large columnar grains with reduced grain boundaries perpendicularly to the thin film surfaces because the oriented columnar grains supported the fast transportation of charge carriers to the selective layers such as the ETL and HTL.^147^

The annealing strategy is the main factor in controlling crystallisation. The close-space annealing strategy (CSA) allowed the grain size to enlarge due to Ostwald ripening and thus enhanced the crystallinity with high-quality optoelectronic properties of APTSCs (narrow bandgap and wide bandgap).^148^

The thickness of the perovskite absorber can control the performance of the PSCs. For a narrow bandgap perovskite (FASnI_3_)_0.6_(MAPbI_3_)_0.4_, 620 nm perovskite thickness was suggested for optimised results.^149^ Thickness also increases with a higher concentration of the perovskite precursor, *e.g.*, a 2.0 M concentration yielded a thickness of 700 nm with 13.1% PCE for a narrow bandgap perovskite (FA_0.75_Cs_0.25_Sn_0.5_Pb_0.5_I_3_).^144^

#### 6.1.2. Bandgap and band alignment

The bandgaps and band alignments are essential for improving performances.

Steric, additive, or compositional engineering can assist in finding suitable bandgaps. The A-site cation can fine-tune the bandgap, while the X-site as part of the metal-halide octahedron can tune the bandgap over a wider range. The dimethylammonium cation (DMA^+^) was reported as a promising option considering the modified tolerance factor of less than 4.18 for the Cs_0.82_DMA_0.18_PbI_3_ composition.^150,151^ DMA^+^ allowed the larger cation to be incorporated before forming a non-photoactive phase. Although the bulky organic molecule led to a decrease in the charge-carrier mobility, lifetime increases from <150 ns to >250 ns.^62^ Another work demonstrated APTSCs by alloying dimethylammonium (DMA^+^) and chloride (Cl^−^) into the perovskite precursor. Using the DMA^+^ and Cl^−^ and reducing Br^−^ in a wide bandgap perovskite, an appropriate bandgap of 1.8 eV was obtained. Simultaneously, the lattice strain and trap densities were minimised. Additionally, this method prevented light-induced halide segregation because the halides were less equimolar.^152^ In a study, antimony potassium tartrate (APTA) was used as an additive in the perovskite precursor expecting multi-functions not only for better band energy alignment but also for suppression of non-radiative recombination and protection against phase segregation utilising the coordination with unbonded lead and blocking the halide migration in perovskites.^29^ FA cation-based perovskite films manifested a narrower bandgap than the MA cation-based ones with 1.31 eV instead of 1.35 eV. The reduction of the bandgap effect was observed in the MA–Sn-based perovskite film (MAPb_0.75_Sn_0.25_I_3_), including the FA cation (MA_0.5_FA_0.5_Pb_0.75_Sn_0.25_I_3_).^143^ FASnI_3_ composition engineering was demonstrated with a standard MAPbI_3_ perovskite precursor (FASnI_3_)_0.6_(MAPbI_3_)_0.4_, with a bandgap of 1.25 eV.^56,149^ A MA-free, FA–Sn-based perovskite film showed a narrower bandgap of ∼1.2 eV compared to a MA–FA–Sn-based perovskite (FA_0.8_MA_0.2_Sn_0.5_Pb_0.5_I_3_) film with a bandgap of 1.26 eV. The MA–free, FA–Sn-based perovskite (FASn_0.5_Pb_0.5_I_3_) can increase the bandgap by incorporating the Cs cation (FA_0.75_Cs_0.25_Sn_0.5_Pb_0.5_I_3_). This strategy improved performance, providing a slightly better-adjusted bandgap, morphology, charge carrier diffusion length and crystal structure.^153,154^

#### 6.1.3. Buffer layer for energy level matching

**Fig. 8:**
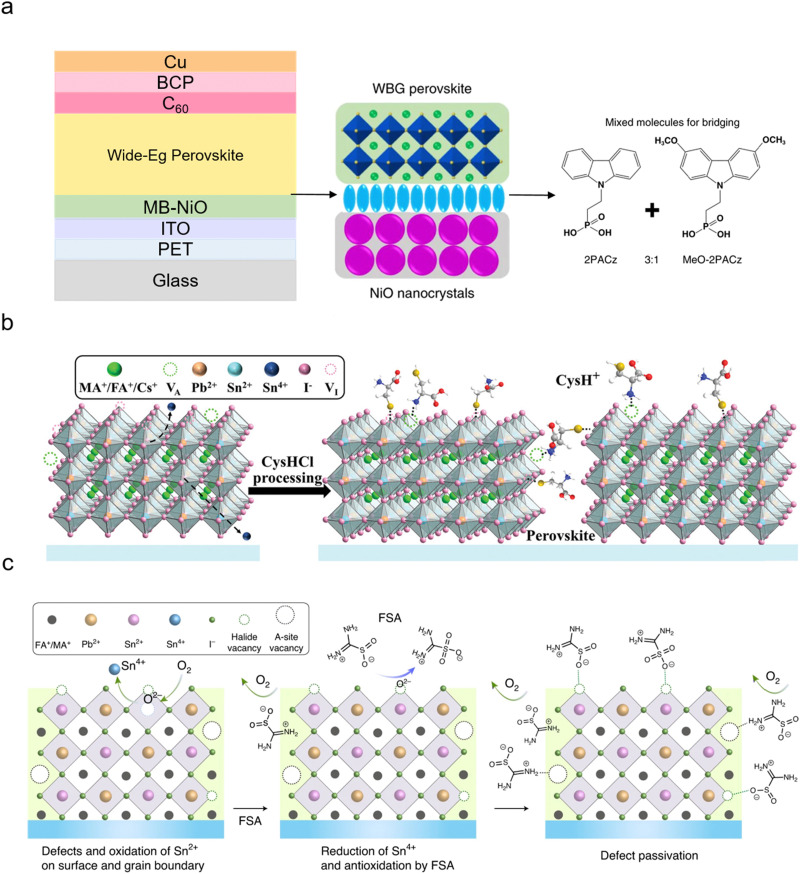
Interfacial type of perovskite film treatment. (a) Bridge structure formed using mixed molecules of 2PACz and MeO-2PACz between a wide bandgap perovskite and a NiO nanocrystal layer. © 2022. Springer Nature All rights reserved.^63^ (b) Illustration of the crystal growth process employing CysHCl in the Sn–Pb perovskite. © 2023. John Wiley and Sons All rights reserved.^26^ (c) Illustration of antioxidation using formamidine sulfinic acid (FSA) in the Sn–Pb perovskite. © 2020. Springer Nature All rights reserved.^53^

Interfacial engineering was also implemented to obtain a favourable energy level. Indene-C_60_-propionic acid hexyl ester (IPH) was used as an interlayer between the perovskite and C_60_, resulting in a conduction band offset of approximately 0.2 eV and a reduced trap density.^155^ Another modification was 1,3-propane diammonium iodide (PDA). PDA suppressed cross-interface recombination by reducing the band offset between C_60_ and the perovskite. It reduced the minority carriers at the interface. Finally, quasi-Fermi-level splitting increased by 90 meV using a wide bandgap of 1.79 eV with *V*_oc_ values of 1.33 V in single junctions and 2.19 V in APTSCs.^156^ Another research demonstrated that ([2-(9*H*-carbazol-9yl)ethyl]phosphonic acid) (2PACz) and ([2-(3,6-dimethoxy-9*H*-carbazol-9-yl)ethyl]phosphonic acid) (MeO-2PACz) were used to bridge the interface. These two chemicals have distinctly different molecular dipole moments. 2PACz and MeO-2PACz molecules anchor on the surface of oxides of NiO, using the phosphonic acid group. This led the molecule-bridged NiO (MB-NiO) to tune the energy-level alignment between NiO and wide bandgap perovskites (Fig. 8a).^63^

#### 6.1.4. Treatment of defects

The defects of the perovskite absorber layer and interface (such as vacancies or grain boundaries) generally deteriorate perovskite crystallinity and affect morphology, diffusion length and recombination leading to low stability and device efficiencies. In order to resolve these defects, mainly two methods were applied. One was additive engineering and the other was (interfacial) passivation engineering.

Due to the oxidation tendency of tin(ii), the Sn-based narrow bandgap perovskite layer had a significant issue with vacancy defects for which various solutions have been proposed. Studies demonstrated potential solutions to solve the unwanted oxidation reaction.^157^ 0.03 mol% of cadmium ions were able to fill the Sn vacancies to de-dope Sn-based perovskites. Although the grain size did not differ from the reference owing to the small amount of cadmium ion, it reinforced electronic properties: the minority carrier recombination lifetime, carrier mobility and diffusion length.^158^ Guanidinium thiocyanate (GuaSCN) has been a popular chemical in single-junction solar cells, and many groups had previously demonstrated high-efficiency devices accordingly.^159,160^ This GuaSCN manifested excellent performances in the tin–lead-based perovskite solar cell (FASnI_3_)_0.6_(MAPbI_3_)_0.4_, improving the optoelectronic properties, reducing energetic disorder and surface recombination velocity, increasing carrier lifetimes, and providing better morphology with suppression of defects in APTSCs.^54,161^ Cysteine hydrochloride (CysHCl) was employed as a bulky passivation and a surface anchoring agent for tin–lead perovskites, reducing trap density and non-radiative recombination and increasing carrier diffusion lengths. This multifunctional organic chemical consists of three functional groups: –NH_3_^+^, –COOH, and –SH. –NH_3_^+^ had the strongest electrostatic potential. –NH_3_^+^ was prone to participate in electrophilic reactions filling the FA^+^/MA^+^/Cs^+^ vacancies. The oxygen and sulfur atoms from –COOH and –SH had lone pair electrons, which coordinated with Sn^2+^ or Pb^2+^, passivating undercoordinated Sn^2+^ or Pb^2+^ and halide vacancies (Fig. 8b).^26^ Use of tin fluoride (SnF_2_) as an additive in Sn–Pb-based perovskites was also studied as a passivation method because the fluorine anion had a strong coordinating ability resulting from its hard Lewis base nature. Therefore, the SnF_2_ additive suppressed tin vacancy formation, creating an SnO_1.2_F(_0.2–0.5_) based interface.^60^ Guanidine tetrafluoroborate (GuaBF_4_) was demonstrated to prohibit the formation of halogen vacancy defects with a minimal effect on the perovskite lattice in an additive method for narrow bandgap perovskites.^132^ Another strategy was to use anchored passivation. The zwitterionic molecules allowed for this surface-anchored passivation. This zwitterionic antioxidant hindered Sn^2+^ oxidation, passivating the surface and improving the uniformity of the film. The formamidine sulfinic acid (FSA) additive acted as a zwitterionic molecule in a tin–lead-based perovskite precursor coordinating to form complexes through dative bonding. This FSA has the ability to passivate both sides of defects, such as electron-donating (FA^+^/MA^+^ vacancies) and electron-accepting (halide vacancies, under-coordinated Pb^2+^/Sn^2+^) (Fig. 8c).^53^ Organic metal coordination compounds can also be employed for additive engineering to make better-quality perovskite films. For example, tris(2,4-pentanedionato)gallium (TPGa) was used for the expected heterovalent substitution and antioxidant effect in Sn-based narrow bandgap perovskites.^162^ A study especially demonstrated utilising the natural SnO_*x*_ for doping in a Sn–Pb-based narrow-bandgap perovskite, resulting in good morphology, defect treatment, and improved carrier recombination. The natural SnO_2_ was produced by exposing the SnI_2_ powder to an oxygen and humid environment and washing it with toluene.^32^

More research on interfacial engineering demonstrated successful treatment of defects. A study reported a quasi-two-dimensional structure (PEA)_2_GAPb_2_I_7_ by adding bulky organics such as phenethylammonium iodide (PEAI) and guanidinium thiocyanate (GASCN) to control defects. This strategy led to improved optoelectronic quality of the Sn–Pb-based perovskite films.^103^ 4-Trifluoromethyl-phenyl ammonium (CF3-PA) was used for the surface treatment by anchoring CF3-PA on the grain surfaces. This mechanism of passivation adsorption stemmed from that of Lewis base molecules. Hence, the perovskite film was prevented from undergoing surface Sn^2+^ oxidation.^163^ In the inorganic-based wide-bandgap perovskite (CsPbI_3−*x*_Br_*x*_), using high-polarity 4-(trifluoromethyl)phenethyl ammonium (CF3-PEA) molecules, a strongly bonding electric dipole interlayer was formed to passivate surface defects and regulate interfacial energy-level alignment, resulting in suppressed non-radiative recombination and improved charge extraction.^131^ A surface-anchored passivating molecule also improved the suppression of Sn^2+^ in a narrow bandgap perovskite. 2-thiopheneethylammonium chloride was used as the 2D perovskite to heal the defects of the perovskite surface. It performed relatively well mitigating interfacial recombination, reducing non-radiative recombination and simultaneously offering band alignment between the perovskite and phenyl-C_60_-butyric acid methyl ester (PCBM).^61^ Using the *n*-butylammonium bromide (BABr), a 2D perovskite was created on the 3D perovskite showing a good open-circuit voltage of 2.33 V.^164^ As another 2D phase passivation layer, lead chloride (PbCl_2_) and phenethylammonium chloride (PMACl) were demonstrated by combining both chemicals. Cl^−^ functioned by decreasing the halide vacancies and suppressing ion migration in the crystal lattice of perovskite film. PMA^+^ facilitated the formation of a 2D perovskite phase on the surface of the perovskite film.^28^ Although the established 2D/3D structure had generally been shown to reduce surface recombination, the approach suffered from transport losses resulting in fill factor reduction. An immiscible 3D/3D bilayer perovskite provided better functions to suppress the interfacial non-radiative recombination and activate charge extraction in the interface between the Sn–Pb-based perovskite and ETL.^5^

Antisolvents were employed for defect passivation as well. When an antisolvent was used, the solvent can simultaneously react with the perovskite bulk and surface, employing a phenethylammonium ligand, *i.e.*, phenethylammonium iodide (PEAI) in ethyl acetate (EA).^165^

### 6.2. HTL (hole transport layer)

In APTSCs, polyethylenedioxythiophene:polystyrenesulfonate (PEDOT:PSS) is a typical choice for the HTL. However, this HTL has challenges including acidity, hygroscopicity, anisotropic charge injection and irreproducibility regarding electrical and physical properties.^166^ Hence, many papers described their own solutions in terms of PEDOT:PSS for APTSCs.

The presence of water in PEDOT:PSS affected the underlying layers, *e.g.* resulting in pinholes and nonuniformity in thin films. The water and PSS-free PEDOT-based HTLs showed good performances with 21.5% efficiency.^167^ In addition, PEDOT:PSS reacted with a tin–lead-based perovskite leading to charge extraction issues during thermal ageing. The indium tin oxide–perovskite heterojunction (without the HTL) showed a good thermal stability of 1000 h at 85 °C, retaining 95% of initial efficiency while suppressing oxidative degradation due to a larger grain size of 350 nm and compactness of tin–lead perovskite films.^120^ Another study showed that simple diluted PEDOT:PSS decreased the work function value in UPS spectra from −4.85 eV to −5.04 eV. This helped energy band alignment improving the performance because of mitigating the unmatched work function.^168^ In recent studies, a ternary Sn(ii) alloy of SnOCl was used as the HTL replacing PEDOT:PSS to obtain bigger grain sizes.^118^ A study demonstrated non-acidic PEDOT:PSS as the HTL because acidic PEDOT:PSS resulted in the formation of I_2_ and quickly oxidised Pb–Sn. Hence using non-acidic PEDOT:PSS, the device showed better thermal stability.^130^

Apart from PEDOT:PSS, other organic or inorganic chemicals were used as HTLs: The *in situ* cross-linked small molecule *N*4,*N*′-di(naphthalen-1-yl)-*N*4,*N*4′-bis(4-vinylphenyl)biphenyl-4,4′-diamine (VNPB) had strong interaction and lower trap density at the interface between VNPB and a wide bandgap perovskite. This resulted in improved device performance with a PCE of 24.9%.^169^ [2-(9*H*-carbazole-9-yl)ethyl] phosphonic acid (2PACz) was employed for wide band gap perovskites to overcome the *V*_oc_ loss at the HTL/perovskite interface reducing the *V*_oc_-deficit of 480 mV at a 1.80 eV bandgap. It resulted in high film uniformity on ITO-based polymer foil.^61^ MeO-2PACz was demonstrated as a HTL reducing the nonradiative losses. This method resulted in a PCE of 24.1% in 2T APTSCs.^27^ Surface treatment using a monomolecular layer, [4-[3-(carbazole-9-yl)carbazole-9-yl]butyl]phosphonic acid (4,3BuPACz) on the poly[bis(4-phenyl)(2,4,6-trimethylphenyl)amine] (PTAA), as a buffer layer for wide bandgap perovskites (single junctions) demonstrated good performance improvement of 16.38% from 14.05% PCE. The nitrogen of the PTAA has a lone pair of electrons as a base. The –P–OH from –PO_3_H_2_ has acidic properties. Therefore, it formed ionic products from the acid and base.^170^ In addition to the molecules mentioned above, 4-(7-(4-(bis(4-methoxyphenyl)amino)-2,5-difluorophenyl)benzo[*c*][1,2,5]thiadiazol-4-yl) benzoic acid (MPA2Ph-BT-BA) and (4-(7*H*-dibenzo[*c*,*g*]carbazole-7-yl)butyl)phosphonic acid (4PADCB) were also used for improving the quality of HTLs.^30,31^

Inorganic HTLs are a good choice for replacing conventional organic HTLs like PEDOT:PSS because of their high thermal stability. The nickel oxide (NiO_*x*_) nanocrystal (NC) is a good candidate due to its dispersibility in water and good thermal stability. The NiO_x_ NC was dissolved in ethanol and used as a HTL in APTSC applications. Ethanol dissolved NiO_*x*_ more than water since ethanol has less intense polarity and protected the previous front subcell during the fabrication. This resulted in improving the thermal stability, *i.e.*, 2500 h at 85 °C, retaining >80% of the initial efficiency.^171^ Another work also demonstrated that NiO_*x*_ is a suitable HTL candidate; NiO_*x*_ was utilised with PTAA as a bilayer. In this configuration, the thick NiO_*x*_ modified the surface of the sub-cells treating defects. This method was used for double and triple junctions.^35^

### 6.3. CRLs (charge recombination layers)

The charge recombination layer (CRL), also called the interconnection layer or tunnel junction, is essential to fabricate a working device in a series connection for a monolithic tandem. To create high-quality CRLs, holes are supposed to transport from the wide bandgap side to the electron transport layer from the narrow bandgap perovskite side. Electrons, conversely, have to be conveyed from the narrow bandgap side to the hole transport layer from the wide bandgap perovskite side. Here, several high-performance cases of CRLs in APTSCs are described.

#### 6.3.1. Initial CRLs

Initial APTSC architecture did not have CRLs. Instead of CRLs, only p-type HTLs and n-type ETLs existed between narrow bandgap perovskites and wide bandgap perovskites as p–n junctions or tunnel junctions. Therefore, there was an issue with decreasing the open-circuit voltage. To solve this problem, research focused on the hole transport material (HTM) layers. First, HTMs such as PTAA or poly(3-hexylthiophene-2,5-diyl) (P3HT) with lithium bis(trifluoromethanesulfonyl)imide (Li-TFSI) and *tert*-butylpyridine (*t*-BP) additives functioned as quasi-solid electrolytes with the properties of conductive hole conductors due to their Li/Li^+^ redox shuttle. These additives led to more conductivity between the HTM and PCBM interface. Hence, the HTM, including additives, can improve the recombination layer quality.^172^ Second, HTM research demonstrated a new type of novel HTL additive. This was the cross-linked p-doped hole transport layer consisting of 1,2-bis[4-(azido-methyl)phenyl]-1,2-diphenylethene (TPE-MN3) and molybdenum tris-[1-(trifluoroethanoyl)-2-(trifluoromethyl)ethane-1,2-dithiolene] (Mo(tfdCOCF_3_)_3_) doped in PTAA. This HTL was a part of the CRL in the APTSC architecture and showed good solvent protection, appropriate energy level and a high electrical conductivity of 8.5 × 10 S cm^−1^ at 1 wt% doping compared to the non-doped one (2.5 × 10^−7^ S cm^−1^). The authors assumed that the azide group would react with reactive nitrene species upon ultraviolet exposure, which was able to be inserted into the C–H bonds in PTAA. It formed insoluble cross-linked networks.^173^

#### 6.3.2. Current CRLs

**Fig. 9:**
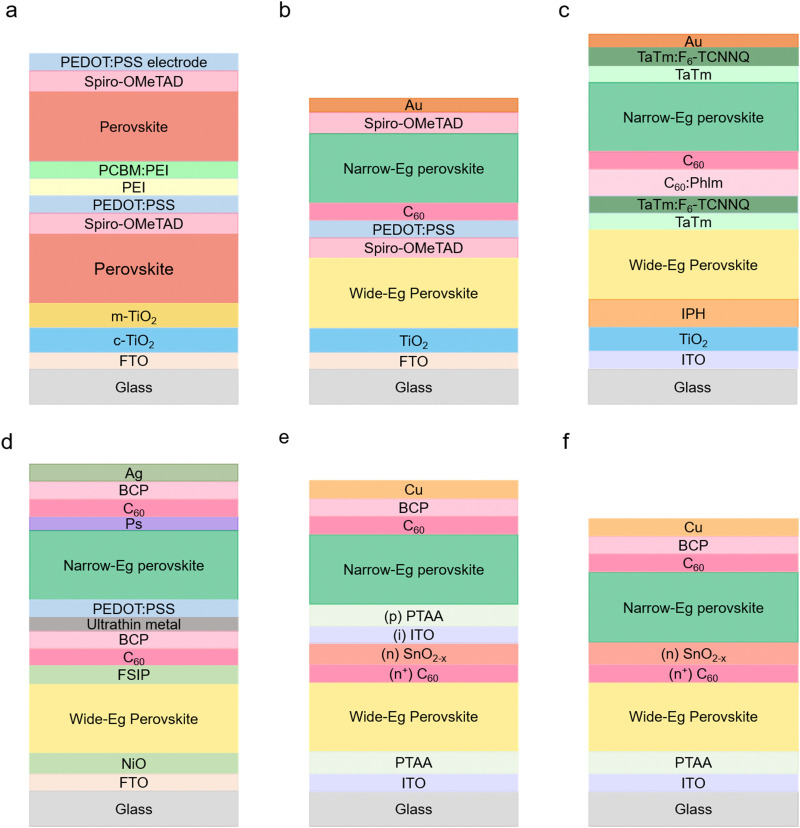
Several configurations of charge recombination layers. (a) Device configuration including the charge recombination layer of spiro-MeOTAD/PEDOT:PSS/PEI (polyethylenimine)/PCBM:PEI. © 2020. Royal Society of Chemistry. All rights reserved.^174^ (b) Cell structure including the charge recombination layer of Spiro-MeOTAD/PEDOT:PSS/C_60_. © 2017. American Chemical Society All rights reserved.^175^ (c) Device structure including an organic dopant (F_6_-TCNNQ) in TaTm for the charge recombination layer configuration. © 2016. John Wiley and Sons. All rights reserved.^176^ (d) Device configuration including the charge recombination layer of (fluoride silane and polyethylenimine ethoxylated (PEIE) in trichloro(3,3,3-trifluoropropyl)silane) FPTS/C_60_/BCP/Cu:Au alloy/PEDOT:PSS. © 2018. John Wiley and Sons. All rights reserved.^177^ (e) Device structure before simplifying the charge recombination layer of C_60_/SnO_2−*x*_/ITO/PTAA. (f) Device structure after simplifying the charge recombination layer of C_60_/SnO_2−*x*_. © 2020. Springer Nature. All rights reserved.^178^

Firstly, the CRL was configured with several layers (spiro-MeOTAD/PEDOT:PSS/PEI(polyethylenimine)/PCBM:PEI) matching with essential functions such as a large work function between the top and bottom surfaces, low-temperature processes, orthogonal solvents, and suitable protective layers for the same solvent (Fig. 9a).^174^ Secondly, another example with several layer configured solution-processed organic-based chemicals (Spiro-MeOTAD/PEDOT:PSS/C_60_) has demonstrated an excellent open-circuit voltage of 1.96 V with high CRL quality. Spiro-MeOTAD and C_60_ acted as p-type and n-type layers, respectively, and thin PEDOT:PSS acted as a recombination layer (Fig. 9b).^175^ Thirdly, organic doping for CRLs is a great option. By doping 2,2′-(perfluoronaphthalene-2,6-diylidene) dimalononitrile (F6-TCNNQ) in *N*4,*N*4,*N*4′′,*N*4′′-tetra([1,1′-biphenyl]-4-yl)-[1,1′:4′,1′′-terphenyl]-4,4′′-diamine (TaTm), TaTm:F_6_-TCNNQ was deposited as a p-doped HTL. In contrast, *N*1,*N*4-bis(tri-*p*-tolylphosphoranylidene) benzene-1,4-diamine (PhIm) was doped in C_60_ as an n-type ETL. These two organic doped layers functioned nicely as CRLs (Fig. 9c).^176^ Fourthly, a thermionic emission-based CRL structure composed of four layers was demonstrated, FSIP/ETL(C_60_/BCP)/ultrathin transparent metal alloy (Cu/Au), and HTL (PEDOT:PSS). Here, FSIP had critical roles in solvent resistance due to its hydrophobicity and good optoelectrical properties. FSIP was considered an ultrathin insulating layer (∼5 nm) because it consisted of fluoride silane and polyethylenimine ethoxylated (PEIE) where trichloro(3,3,3-trifluoropropyl)silane (FPTS) was proposed as a silanising agent incorporated on PEIE (Fig. 9d).^177^ Lastly, a study has successfully tested the simplification of a CRL because the conventional CRL consisted of four or more layers. The layers for CRLs had issues regarding device performance. For example, ITO, indium-doped zinc oxide (IZO) or gold resulted in current density losses due to parasitic absorption and reflection at the interface forming the multilayers, while the sputtering deposition processes damaged the underlying layers, reducing the fill factor. A complex to simple CRL was achieved using the C_60_/SnO_1.76_ layer. In the fabrication process, C_60_ was fortuitously n-doped by iodine ions from the previous perovskite layer, which functioned as an effective electron-selective layer. The incomplete oxidation of tin had the property of ambipolar carrier transport due to the large density of Sn^2+^ (Fig. 9e and f).^178^

### 6.4. Electrodes

In 4T-based APTSCs, the research regarding electrodes on the HTL in wide bandgap-based perovskite solar cells is necessary owing to the optoelectrical losses. The conventional MoO_*x*_/Au/MoO_*x*_ electrode limited the transmittance due to parasitic absorption from the thin metal layer. Replacing MoO_*x*_/Au/MoO_*x*_ with transparent MoO_*x*_/indium tin oxide (ITO) led to an improved transmittance in the infrared range. Consequently, the light transparency of the infrared range in the top cell was enhanced up to 70%.^179^ Another electrode study used highly transparent hydrogen-doped indium oxide (IO:H) to replace ITO as the electrode, which was an option with the HTL-free configuration in 4T APTSCs. IO:H improved photocurrent, thus reducing ultra-low near-infrared optical loss and increasing high charge carrier mobility with the HTL-free configuration.^180^

## 7. Simulated models of APTSCs

Since 2017, the number of simulation models has increased attempting a full APTSC model.^205^ Many simulations have attempted to optimise the thickness of the layers. Another increasing trend is the simulation of lead-free perovskite materials. Here, the research in terms of thickness simulation with lead and without lead is briefly described.

### 7.1. Lead-containing perovskite simulations

One simulation study derived that the ideal thickness of the subcells was 350 nm, reaching the maximum output efficiency of 36.6%, with the bandgap of the top cell being 1.5 eV and the bottom cell bandgap being 0.95 eV.^206^ Another study introduced CsPbIBr_2_ at 2.05 eV for the top cell and MAPbI_3_ at 1.55 eV for the bottom cell. The optimised thickness was 600 nm for the top cell and 500 nm for the properties of CsPbIBr_2_. A maximum PCE of 27.4% was calculated.^207^

### 7.2. Lead-free perovskite simulations

Although Pb-based PSCs are a promising state-of-the-art technology in the field of photovoltaics, due to lead toxicity, many groups are interested in lead-free perovskites. Concerns are focused on the leakage of lead from broken modules due to natural disasters/phenomena such as earthquakes, hail or heavy rain, even though the overall lead content of PSCs is low at 0.4 g m^−2^.^208^ Thus, many simulations were conducted to study possible lead-free alternatives.

There are several papers regarding the thickness optimisation of lead-free perovskite absorbers. One work simulated a perovskite with a wide bandgap (Cs_2_AgBi_0.75_Sb_0.25_Br_6_) of 1.8 eV and a low Pb content-based perovskite (FACsPb_0.5_Sn_0.5_I_3_) with a narrow bandgap of 1.2 eV. In the APTSC configuration, the optimised thicknesses of the wide bandgap and the narrow band gap perovskites are 380 nm and 400 nm, respectively, reaching the PCE of 17.35%.^209^ When the MASnI_3_ perovskite with a narrow bandgap of 1.3 eV and Cs_2_AgBi_0.75_Sb_0.25_Br_6_ with a wide bandgap of 1.8 eV were employed, the simulation indicated that a maximum PCE of 24.86% can be achieved.^210^ Another work utilised germanium to replace Pb for achieving a wide bandgap. A combination of methyl ammonium germanium halide was produced with a wide bandgap of 1.9 eV. Germanium slightly increased the narrow bandgap (FA_0.75_MA_0.25_Sn_0.25_Ge_0.5_I_3_), reaching the bandgap of 1.4 eV. The optimised APTSC efficiency was 26.72% considering the different ETLs such as SnO_2_, IGZO, PCBM and ZnO.^211^ Another simulation also employed germanium in the perovskite composition (CsSn_0.5_Ge_0.5_I_3_) for achieving a wide bandgap of 1.5 eV. In contrast, only Sn was used instead of tin–lead mixed perovskite solution showing 1.3 eV for a narrow bandgap perovskite. The optimised perovskite thickness of 2T is 450 nm and 812 nm for wide and narrow bandgap perovskites, respectively, achieving a PCE of 18.32%. On the other hand, for 4T APTSCs, optimised perovskite thickness was thicker than the thickness of 2T at 1300 nm and 900 nm for wide and narrow bandgaps showing a PCE of 19.86%.^212^ Another simulation used germanium for lead-free APTSCs as an alternative to lead. The optimised thickness of the wide bandgap (MAGeI_3_) was 983 nm, and the thickness of the narrow bandgap was 1600 nm. With this structure, the calculation showed a PCE of 30.85%.^213^

## 8. Conclusion and perspectives

Many state-of-the-art PV technologies have gradually progressed from single-junction solar cells to double or triple-junction solar cells. The increasing attention on the APTSCs has been proven by the number of published papers annually and the improved device performances. In this review, we introduced the fundamentals of APTSCs, the approaches for the current issues, and the potential of APTSCs in the future.

The tandem technology is one of the key methods beyond the single junction S–Q limit. The tunable bandgap of the perovskite provides the privilege of high efficiency towards ideally 45% instead of 33%. Additionally, the low cost originating from the thin film technology, lightweight, scale-up chances, and eco-friendly technology related to a low CO_2_ footprint are highly attractive.

We elaborated on various types of APTSCs considering 2T and 4T architectures and classified APTSCs into bifacial, inorganic, flexible substrate-based, substrate configured and multi-junction APTSCs beyond the double junctions.

However, these high-tech APTSCs faced several issues regarding stability caused by Sn^2+^ oxidation of narrow bandgap perovskites, *V*_oc_ loss of wide bandgap perovskites, non-standardisation of CRLs, and inhomogeneity in the layers during the deposition of a multilayer stack. We also offered information regarding each layer of the APTSC configuration and solutions. Most of the resolutions consisted of using additives or passivation processes for controlling the crystal growth rate, managing bandgaps and band alignment, and treating the defects.

Furthermore, to show the potential of the APTSCs, we summarised the simulation studies. A variety of simulations were implemented with diverse APTSC conditions. Mainly, the simulation research demonstrated the possibility of improving the performance of the APTSCs beyond 30%.

In the journey to commercialising APTSCs, we suggest possible milestones to achieve PCEs >30% with all-perovskite tandem modules.

Blade-coating (instead of spin-coating) methods are currently demonstrated for the scale-up. In addition, slot-die coating, spray coating, inkjet printing, and screen printing also have the potential towards scaling APTSCs.

Furthermore, we need more studies on the flexible substrate-based APTSCs for mass production. The roll-to-roll process can accelerate perovskite market growth.

To develop high-performance scaled APTSCs, we consider three main engineering aspects: buried interface engineering, simplifying the charge recombination layer, and reducing the vacuum process. The first technical method is buried interface engineering (so-called additive engineering), which provides several advantages. Buried interface engineering enables fast device manufacturing speed as it does not demand an extra-thin passivation layer. Additionally, it does not demand high-quality super-thin layer technology for the passivation layers. It can not only improve the quality of the perovskite, ETL, or HTL bulk layer but also achieve the passivation effect.^58,189,197,201^ The second technical method is to simplify the charge recombination layer. The complicated structure of the charge recombination layer constructed using different deposition techniques can retard the commercialisation of APTSCs regarding the design of the manufacturing execution system (MES). The third technical method is to reduce the vacuum process as much as possible during device fabrication. Although using atomic layer deposition or sputtering deposition can provide excellent layers, as mentioned above, the different deposition techniques can hinder production efficiency. Moreover, a vacuum can be detrimental to perovskite solar cells, affecting their device lifetime.^230,231^

However, considering the low durability of perovskites against humidity and oxygen, a plant may require an inert gas system (or an otherwise controlled environment) until encapsulation is applied.

In addition, for commercialising APTSCs, highly efficient and stable all-perovskite module technologies are crucial, such as cell-to-module (CTM) technology. In this case, interconnection development will be necessary to minimise dead areas, thus increasing the geometric fill factors to reduce CTM loss.

## Author contributions

All authors contributed to the discussion of this content. J. L. wrote the initial manuscript. N. P., S. S. and M. S. revised and edited the manuscripts before submission.

## Conflicts of interest

There are no conflicts to declare.

## Supplementary Material

EE-017-D3EE03638C-s001
