# Supplementary material for: All-perovskite tandem solar cells: from fundamentals to technological progress
Source: Energy Environ Sci. 2024 May 10;17(13):4390–425. doi: 10.1039/d3ee03638c (PMC11218037; doi:10.1039/d3ee03638c)
Supplement: EE-017-D3EE03638C-s001 [file EE-017-D3EE03638C-s001.pdf]

Electronic Supplementary Material (ESI) for Energy & Environmental Science.
This journal is © The Royal Society of Chemistry 2024

All-perovskite tandem solar cells from fundamentals to technological progress

Jaekeun Lim[a], Nam-Gyu Park*[b,c], Sang Il Seok*[d] and Michael Saliba*[a,e]

[a.] Institute for Photovoltaics (ipv), University of Stuttgart, Stuttgart, Germany
[b.] School of Chemical Engineering and Center for Antibonding Regulated Crystals, Sungkyunkwan University, Suwon, Republic of Korea
[c.] SKKU Institute of Energy Science and Technology (SIEST), Sungkyunkwan University, Suwon, Republic of Korea
[d.] Department of Energy Engineering, School of Energy and Chemical Engineering, Ulsan National Institute of Science and Technology, Ulsan, South Korea
[e.] Helmholtz Young Investigator Group FRONTRUNNER, IEK5-Photovoltaik, Forschungszentrum Jülich, Jülich, Germany

Supporting Information Table 1. Device information of Fig. 1a-d.

*Ref. 196 is not shown in Fig. 1b as Ref. 196 is overlapped with Ref. 163.

Tandem PCE (%)	Active area (mm^2)	Terminal	Note	Ref.
28.5<br>28	4.9<br>4.9	2T<br>2T	Fig. 1a<br>Certified	5
25.7	5.76	2T	Certified	26
26.3	5.76	2T	Certified	30
26.4	104.4	2T	Certified	31
25.1<br>23.8	4.9<br>4.9	2T<br>2T	Triple junction<br>Triple junction & Certified	33
24.3<br>23.2	4.9<br>4.9	2T<br>2T	Fig. 1a & Triple junction<br>Triple junction & Certified	34
6.7	9.19	2T	Fig. 1a & Triple junction	35
20.1	4.9	2T	Fig. 1a & Triple junction	36
16.8	6.76	2T	Triple junction	37
25.6<br>24.2	4.9<br>100	2T<br>2T	Fig. 1a<br>Certified	53
15.3	5000	4T	Flexible & Module	54
19.1<br>19.8<br>18.3	1225<br>243<br>243	2T<br>2T<br>2T	Module<br>Module<br>Module & Certified	55
22.5<br>21.7	2025<br>2025	2T<br>2T	Module<br>Module & Certified	56
21.6	1430	2T	Module	57
24.5<br>24.9<br>23.8	2025<br>2000<br>6400	2T<br>2T<br>2T	Certified & Module<br>Module<br>Module	58
22.6<br>23.8	9<br>9	4T<br>2T	Flexible substrate<br>Flexible substrate	61
21.3	5.8	2T	Flexible substrate	62
24.4	4.9	2T	Certified & Flexible substrate	63
24.3	9	2T	Certified	103
28.51	9	2T	Bifacial	128
29.3	8	2T	Bifacial	130
24.1<br>20.3	5.29<br>5.29	2T<br>2T	Flexible substrate<br>Flexible substrate	132
19.08	3.14 or 10	4T	Fig. 1a	143

24.79	9	2T	Certified	148
21.2	8.5	4T	Fig. 1a	149
20.1	20	4T	Fig. 1a	153
27.4<br>26.29	4.9<br>4.9	2T<br>2T	Fig. 1a<br>Certified	156
24.79<br>22	7.3<br>105	2T<br>2T	Certified<br>Certified	157
25.4	10.5	4T	Fig. 1a	161
26.7<br>26.4	4.9<br>4.9	2T<br>2T	Fig. 1a<br>Certified	163
10.8	9.6	2T	Fig. 1a	172
23.1	10	4T	Fig. 1a	179
23.3	2025	4T	Module	188
27.5	12	2T	Certified	190
26.96	4.2	2T	Certified	193
22.2	9	2T	Flexible substrate	195
26.4	4.9	2T	Certified	196*
27.04	4.2	2T	Certified	199

Supporting Information Table 2.A. Absorber layer thickness database of double junction APTSCs.

WB*: wide bandgap

NB*: narrow bandgap

Thickness data was extracted manually from each paper's Scanning Electron Microscope (SEM) images.

§: requested to author to obtain the value

Max. WB thickness (nm)	Min. WB thickness (nm)	Max. NB thickness (nm)	Min. NB thickness (nm)	Tandem PCE (%)	Termina l	Ref.
382	319	1277	1172	28.5	2T	5
378	296	922	768	25.7	2T	26
378	318	788	711	26.68	2T	28
774	719	N/A	N/A	26.3	4T	29
304	230	646	484	27.22	2T	30
268	243	726	694	27	2T	31
279	243	730	905	20.1	2T	36
530	477	1036	819	25.6	2T	53
211	125	436	374	15.3	4T	54
345	279	892	792	25.1	2T	56
248	183	1041	854	23.1	2T	57
298	246	973	897	26.8	2T	58
ca. 500§	ca. 500§	594	500	23.1	4T	60
340	281	781	630	23.8<br>22.6	2T<br>4T	61
333	269	861	691	23.1	2T	62
533	370	963	823	24.7	2T	63
424	408	1058	894	25.5	2T	103
N/A	N/A	536	439	20.8	2T	120
389	336	994	884	26.3	2T	121
480	365	1083	973	28.51	2T	128
434	430	917	700	20.1	2T	129
607	558	1889	806	24.4 (Mono)<br>29.3 (Bifacial)	2T	130
360	240	957	742	25.6	2T	131

353	261	940	895	25.3	2T	132
159	130	160	103	19.08	2T	143
473	388	727	669	19.1	2T	144
277	251	805	716	21.1	2T	145
273	255	713	593	10.5	2T	146
N/A	N/A	529	396	23	4T	147
230	200	628	567	25.15	2T	148
N/A	N/A	672	618	21.2	4T	149
360	325	1040	962	26.2	2T	152
461	343	470	332	16.9	2T	153
N/A	N/A	559	504	23.6	4T	154
488	351	1055	928	27.4	2T	156
353	271	702	543	24.79	2T	157
471	421	960	727	23	2T	158
N/A	N/A	1016	855	23.1<br>25.4	2T<br>4T	161
633	581	N/A	N/A	23.1	4T	162
452	321	1145	1133	26.7	2T	163
259	205	607	534	23.7	2T	165
321	272	700	690	21.5	2T	167
321	279	877	750	24.9	2T	169
477	388	776	715	23.5	2T	171
588	427	260	229	10.4	2T	172
437	261	161	114	5.1	2T	175
290	244	252	212	17.9	2T	177
461	366	726	636	24.4	2T	178
212	160	368	336	18.4	2T	181
271	236	703	629	24.1	2T	182
478	379	865	755	24.66	2T	183
424	384	884	687	26.08	2T	184
512	484	N/A	N/A	28.06	4T	186
411	339	857	753	26.03	2T	187
424	342	1085	972	23.3	4T	188
425	314	564	385	23.52	2T	189
366	311	912	803	27.5	2T	190
243	291	857	693	26.1	2T	192
380	362	825	658	27.3	2T	193
530	436	N/A	N/A	27.64	4T	194
372	361	1248	949	26.1	2T	195
540	352	1100	784	27.3	2T	196
393	382	1123	876	27.1	2T	198
402	312	974	867	27.04	2T	199
393	388	848	768	26.33	2T	200
678	563	N/A	N/A	28.35	4T	201
436	330	1060	928	27	2T	202
363	306	-	-	26.89	4T	203
435	420	-	-	28.07	4T	204

Supporting Information Table 2.B. Absorber layer thickness database of triple junction APTSCs.

WB*: wide bandgap

IB*: intermediate bandgap

NB*: narrow bandgap

Thickness data was extracted manually from each paper's SEM images.

Max. WB thickness	Min. WB thickness	Max. IB thickness	Min. IB thickness	Max. NB thickness	Min. NB thickness	Tandem PCE (%)	Terminal	Ref.

(nm)	(nm)	(nm)	(nm)	(nm)	(nm)			
236	195	1081	1020	1236	1126	24.3	2T	34
502	467	654	567	473	368	6.7	2T	35
96	80	367	327	464	412	16.8	2T	37

References

5 R. Lin, Y. Wang, Q. Lu, B. Tang, J. Li, H. Gao, Y. Gao, H. Li, C. Ding, J. Wen, P. Wu, C. Liu, S. Zhao, K. Xiao, Z. Liu, C. Ma, Y. Deng, L. Li, F. Fan and H. Tan, Nature, , DOI:10.1038/s41586-023-06278-z.

26 J. Luo, R. He, H. Lai, C. Chen, J. Zhu, Y. Xu, F. Yao, T. Ma, Y. Luo, Z. Yi, Y. Jiang, Z. Gao, J. Wang, W. Wang, H. Huang, Y. Wang, S. Ren, Q. Lin, C. Wang, F. Fu and D. Zhao, Advanced Materials, 2023, 2300352.

28 Y. Zhao, C. Wang, T. Ma, L. Zhou, Z. Wu, H. Wang, C. Chen, Z. Yu, W. Sun, A. Wang, H. Huang, B. Zou, D. Zhao and X. Li, Energy Environ Sci, , DOI:10.1039/d2ee04087e.

29 X. Hu, J. Li, C. Wang, H. Cui, Y. Liu, S. Zhou, H. Guan, W. Ke, C. Tao and G. Fang, Nanomicro Lett, , DOI:10.1007/s40820-023-01078-6.

30 J. Zhu, Y. Luo, R. He, C. Chen, Y. Wang, J. Luo, Z. Yi, J. Thiesbrummel, C. Wang, F. Lang, H. Lai, Y. Xu, J. Wang, Z. Zhang, W. Liang, G. Cui, S. Ren, X. Hao, H. Huang, Y. Wang, F. Yao, Q. Lin, L. Wu, J. Zhang, M. Stolterfoht, F. Fu and D. Zhao, Nat Energy, , DOI:10.1038/s41560-023-01274-z.

31 R. He, W. Wang, Z. Yi, F. Lang, C. Chen, J. Luo, J. Zhu, J. Thiesbrummel, S. Shah, K. Wei, Y. Luo, C. Wang, H. Lai, H. Huang, J. Zhou, B. Zou, X. Yin, S. Ren, X. Hao, L. Wu, J. Zhang, J. Zhang, M. Stolterfoht, F. Fu, W. Tang and D. Zhao, Nature, , DOI:10.1038/s41586-023-05992-y.

33 J. Wang, L. Zeng, D. Zhang, A. Maxwell, H. Chen, K. Datta, A. Caiazzo, W. H. M. Remmerswaal, N. R. M. Schipper, Z. Chen, K. Ho, A. Dasgupta, G. Kusch, R. Ollearo, L. Bellini, S. Hu, Z. Wang, C. Li, S. Teale, L. Grater, B. Chen, M. M. Wienk, R. A. Oliver, H. J. Snaith, R. A. J. Janssen and E. H. Sargent, Nat Energy, , DOI:10.1038/s41560-023-01406-5.

34 Z. Wang, L. Zeng, T. Zhu, H. Chen, B. Chen, D. J. Kubicki, A. Balvanz, C. Li, A. Maxwell, E. Ugur, R. dos Reis, M. Cheng, G. Yang, B. Subedi, D. Luo, J. Hu, J. Wang, S. Teale, S. Mahesh, S. Wang, S. Hu, E. D. Jung, M. Wei, S. M. Park, L. Grater, E. Aydin, Z. Song, N. J. Podraza, Z. H. Lu, J. Huang, V. P. Dravid, S. De Wolf, Y. Yan, M. Grätzel, M. G. Kanatzidis and E. H. Sargent, Nature, 2023, 618, 74–79.

35 D. P. McMeekin, S. Mahesh, N. K. Noel, M. T. Klug, J. C. Lim, J. H. Warby, J. M. Ball, L. M. Herz, M. B. Johnston and H. J. Snaith, Joule, 2019, 3, 387–401.

36 K. Xiao, J. Wen, Q. Han, R. Lin, Y. Gao, S. Gu, Y. Zang, Y. Nie, J. Zhu, J. Xu and H. Tan, ACS Energy Lett, 2020, 5, 2819–2826.

37 J. Wang, V. Zardetto, K. Datta, D. Zhang, M. M. Wienk and R. A. J. Janssen, Nat Commun, , DOI:10.1038/s41467-020-19062-8.

53 K. Xiao, R. Lin, Q. Han, Y. Hou, Z. Qin, H. T. Nguyen, J. Wen, M. Wei, V. Yeddu, M. I. Saidaminov, Y. Gao, X. Luo, Y. Wang, H. Gao, C. Zhang, J. Xu, J. Zhu, E. H. Sargent and H. Tan, Nat Energy, 2020, 5, 870–880.

54 V. Babu, M. A. Mejia Escobar, R. Fuentes Pineda, M. Ścigaj, P. Spinelli and K. Wojciechowski, Mater Today Energy, , DOI:10.1016/j.mtener.2022.101073.

55 B. Abdollahi Nejand, D. B. Ritzer, H. Hu, F. Schackmar, S. Moghadamzadeh, T. Feeney, R. Singh, F. Laufer, R. Schmager, R. Azmi, M. Kaiser, T. Abzieher, S. Gharibzadeh, E. Ahlswede, U. Lemmer, B. S. Richards and U. W. Paetzold, Nat Energy, 2022, 7, 620–630.

56 K. Xiao, Y.-H. Lin, M. Zhang, R. D. J. Oliver, X. Wang, Z. Liu, X. Luo, J. Li, D. Lai, H. Luo, R. Lin, J. Xu, Y. Hou, H. J. Snaith and H. Tan, Scalable processing for realizing 21.7%-efficient all-perovskite tandem solar modules, 2022, vol. 376.

57 X. Dai, S. Chen, H. Jiao, L. Zhao, K. Wang, Z. Ni, Z. Yu, B. Chen, Y. Gao and J. Huang, Nat Energy, 2022, 7, 923–931.

58 H. Gao, K. Xiao, R. Lin, S. Zhao, W. Wang, S. Dayneko, C. Duan, C. Ji, H. Sun, A. Dinh Bui, C. Liu, J. Wen, W. Kong, H. Luo, X. Zheng, Z. Liu, H. Nguyen, J. Xie, L. Li, M. I. Saidaminov and H. Tan, Homogeneous crystallization and buried interface passivation for perovskite tandem solar modules, .

60 J. Kurisinkal Pious, Y. Zwirner, H. Lai, S. Olthof, Q. Jeangros, E. Gilshtein, R. K. Kothandaraman, K. Artuk, P. Wechsler, C. Chen, C. M. Wolff, D. Zhao, A. N. Tiwari and F. Fu, ACS Appl Mater Interfaces, , DOI:10.1021/acsami.2c19124.

61 H. Lai, J. Luo, Y. Zwirner, S. Olthof, A. Wieczorek, F. Ye, Q. Jeangros, X. Yin, F. Akhundova, T. Ma, R. He, R. K. Kothandaraman, X. Chin, E. Gilshtein, A. Müller, C. Wang, J. Thiesbrummel, S. Siol, J. M. Prieto, T. Unold, M. Stolterfoht, C. Chen, A. N. Tiwari, D. Zhao and F. Fu, Adv Energy Mater, , DOI:10.1002/aenm.202202438.

62 A. F. Palmstrom, G. E. Eperon, T. Leijtens, R. Prasanna, S. N. Habisreutinger, W. Nemeth, E. A. Gaulding, S. P. Dunfield, M. Reese, S. Nanayakkara, T. Moot, J. Werner, J. Liu, B. To, S. T. Christensen, M. D. McGehee, M. F. A. M. van Hest, J. M. Luther, J. J. Berry and D. T. Moore, Joule, 2019, 3, 2193–2204.

63 L. Li, Y. Wang, X. Wang, R. Lin, X. Luo, Z. Liu, K. Zhou, S. Xiong, Q. Bao, G. Chen, Y. Tian, Y. Deng, K. Xiao, J. Wu, M. I. Saidaminov, H. Lin, C. Q. Ma, Z. Zhao, Y. Wu, L. Zhang and H. Tan, Nat Energy, 2022, 7, 708–717.

103 J. Tong, Q. Jiang, A. J. Ferguson, A. F. Palmstrom, X. Wang, J. Hao, S. P. Dunfield, A. E. Louks, S. P. Harvey, C. Li, H. Lu, R. M. France, S. A. Johnson, F. Zhang, M. Yang, J. F. Geisz, M. D. McGehee, M. C. Beard, Y. Yan, D. Kuciauskas, J. J. Berry and K. Zhu, Nature Energy 2022 7:7, 2022, 7, 642–651.

120 R. Prasanna, T. Leijtens, S. P. Dunfield, J. A. Raiford, E. J. Wolf, S. A. Swifter, J. Werner, G. E. Eperon, C. de Paula, A. F. Palmstrom, C. C. Boyd, M. F. A. M. van Hest, S. F. Bent, G. Teeter, J. J. Berry and M. D. McGehee, Nat Energy, 2019, 4, 939–947.

121 P. Wu, J. Wen, Y. Wang, Z. Liu, R. Lin, H. Li, H. Luo and H. Tan, Adv Energy Mater, , DOI:10.1002/aenm.202202948.

128 H. Li, Y. Wang, H. Gao, M. Zhang, R. Lin, P. Wu, K. Xiao and H. Tan, eLight, , DOI:10.1186/s43593-022-00028-w.

129 L. Gil-Escrig, S. Hu, K. P. S. Zanoni, A. Paliwal, M. A. Hernández-Fenollosa, C. Roldán-Carmona, M. Sessolo, A. Wakamiya and H. J. Bolink, ACS Mater Lett, 2022, 4, 2638–2644.

130 B. Chen, Z. Yu, A. Onno, Z. Yu, S. Chen, J. Wang, Z. C. Holman and J. Huang, Bifacial all-perovskite tandem solar cells, 2022, vol. 8.

131 T. Li, J. Xu, R. Lin, S. Teale, H. Li, Z. Liu, C. Duan, Q. Zhao, K. Xiao, P. Wu, B. Chen, S. Jiang, S. Xiong, H. Luo, S. Wan, L. Li, Q. Bao, Y. Tian, X. Gao, J. Xie, E. H. Sargent and H. Tan, Nat Energy, , DOI:10.1038/s41560-023-01250-7.

132 Y. Wang, R. Lin, X. Wang, C. Liu, Y. Ahmed, Z. Huang, Z. Zhang, H. Li, M. Zhang, Y. Gao, H. Luo, P. Wu, H. Gao, X. Zheng, M. Li, Z. Liu, W. Kong, L. Li, K. Liu, M. I. Saidaminov, L. Zhang and H. Tan, Nat Commun, 2023, 14, 1819.

143 Z. Yang, A. Rajagopal, C. C. Chueh, S. B. Jo, B. Liu, T. Zhao and A. K. Y. Jen, Advanced Materials, 2016, 28, 8990–8997.

144 T. Leijtens, R. Prasanna, K. A. Bush, G. E. Eperon, J. A. Raiford, A. Gold-Parker, E. J. Wolf, S. A. Swifter, C. C. Boyd, H. P. Wang, M. F. Toney, S. F. Bent and M. D. McGehee, Sustain Energy Fuels, 2018, 2, 2450–2459.

145 Z. Song, D. Zhao, C. Chen, R. H. Ahangharnejhad, C. Li, K. Ghimire, N. J. Podraza, M. J. Heben, K. Zhu and Y. Yan, in 2019 IEEE 46th Photovoltaic Specialists Conference (PVSC), 2019, pp. 743–746.

146 D. Zhao, C. Chen, C. Wang, M. M. Junda, Z. Song, C. R. Grice, Y. Yu, C. Li, B. Subedi, N. J. Podraza, X. Zhao, G. Fang, R. G. Xiong, K. Zhu and Y. Yan, Nat Energy, 2018, 3, 1093–1100.

147 B. Abdollahi Nejand, I. M. Hossain, M. Jakoby, S. Moghadamzadeh, T. Abzieher, S. Gharibzadeh, J. A. Schwenzer, P. Nazari, F. Schackmar, D. Hauschild, L. Weinhardt, U. Lemmer, B. S. Richards, I. A. Howard and U. W. Paetzold, Adv Energy Mater, , DOI:10.1002/aenm.201902583.

148 C. Wang, Y. Zhao, T. Ma, Y. An, R. He, J. Zhu, C. Chen, S. Ren, F. Fu, D. Zhao and X. Li, Nat Energy, 2022, 7, 744–753.

149 D. Zhao, Y. Yu, C. Wang, W. Liao, N. Shrestha, C. R. Grice, A. J. Cimaroli, L. Guan, R. J. Ellingson, K. Zhu, X. Zhao, R. G. Xiong and Y. Yan, Nat Energy, , DOI:10.1038/nenergy.2017.18.

152 J. Wen, Y. Zhao, Z. Liu, H. Gao, R. Lin, S. Wan, C. Ji, K. Xiao, Y. Gao, Y. Tian, J. Xie, C. J. Brabec and H. Tan, Advanced Materials, , DOI:10.1002/adma.202110356.

153 G. E. Eperon, T. Leijtens, K. A. Bush, R. Prasanna, T. Green, J. T.-W. Wang, D. P. McMeekin, G. Volonakis, R. L. Milot, R. May, A. Palmstrom, D. J. Slotcavage, R. A. Belisle, J. B. Patel, E. S. Parrott, R. J. Sutton, W. Ma, F. Moghadam, B. Conings, A. Babayigit, H.-G. Boyen, S. Bent, F. Giustino, L. M. Herz, M. B. Johnston, M. D. McGehee and H. J. Snaith, Science (1979), 2016, 354, 861–865.

154 S. Moghadamzadeh, I. M. Hossain, T. Duong, S. Gharibzadeh, T. Abzieher, H. Pham, H. Hu, P. Fassl, U. Lemmer, B. A. Nejand and U. W. Paetzold, J Mater Chem A Mater, 2020, 8, 24608–24619.

156 H. Chen, A. Maxwell, C. Li, S. Teale, B. Chen, T. Zhu, E. Ugur, G. Harrison, L. Grater, J. Wang, Z. Wang, L. Zeng, S. M. Park, L. Chen, P. Serles, R. A. Awni, B. Subedi, X. Zheng, C. Xiao, N. J. Podraza, T. Filleter, C. Liu, Y. Yang, J. M. Luther, S. De Wolf, M. G. Kanatzidis, Y. Yan and E. H. Sargent, Nature, 2023, 613, 676–681.

157 R. Lin, K. Xiao, Z. Qin, Q. Han, C. Zhang, M. Wei, M. I. Saidaminov, Y. Gao, J. Xu, M. Xiao, A. Li, J. Zhu, E. H. Sargent and H. Tan, Nat Energy, 2019, 4, 864–873.

158 Z. Yang, Z. Yu, H. Wei, X. Xiao, Z. Ni, B. Chen, Y. Deng, S. N. Habisreutinger, X. Chen, K. Wang, J. Zhao, P. N. Rudd, J. J. Berry, M. C. Beard and J. Huang, Nat Commun, , DOI:10.1038/s41467-019-12513-x.

161 J. Tong, Z. Song, D. Hoe Kim, X. Chen, C. Chen, A. F. Palmstrom, P. F. Ndione, M. O. Reese, S. P. Dunfield, O. G. Reid, J. Liu, F. Zhang, S. P. Harvey, Z. Li, S. T. Christensen, G. Teeter, D. Zhao, M. M. Al-Jassim, M. F. A M van Hest, M. C. Beard, S. E. Shaheen, J. J. Berry, Y. Yan and K. Zhu, Carrier lifetimes of >1 ms in Sn-Pb perovskites enable efficient all-perovskite tandem solar cells, 2019, vol. 364.

162 Z. Chang, D. Zheng, S. Zhao, L. Wang, S. Wu, L. Liu, Z. Li, L. Zhang, Q. Dong, H. Wang, S. Wang, K. Wang and S. Liu, Adv Funct Mater, , DOI:10.1002/adfm.202214983.

163 R. Lin, J. Xu, M. Wei, Y. Wang, Z. Qin, Z. Liu, J. Wu, K. Xiao, B. Chen, S. M. Park, G. Chen, H. R. Atapattu, K. R. Graham, J. Xu, J. Zhu, L. Li, C. Zhang, E. H. Sargent and H. Tan, Nature, 2022, 603, 73–78.

165 M. Wei, K. Xiao, G. Walters, R. Lin, Y. Zhao, M. I. Saidaminov, P. Todorović, A. Johnston, Z. Huang, H. Chen, A. Li, J. Zhu, Z. Yang, Y. K. Wang, A. H. Proppe, S. O. Kelley, Y. Hou, O. Voznyy, H. Tan and E. H. Sargent, Advanced Materials, , DOI:10.1002/adma.201907058.

167 M. A. Mahmud, J. Zheng, S. Tang, C. Liao, G. Wang, J. Bing, T. L. Leung, A. D. Bui, H. Chen, J. Yi, S. P. Bremner, H. T. Nguyen and A. W. Y. Ho-Baillie, ACS Energy Lett, 2023, 8, 21–30.

169 Y. Wang, S. Gu, G. Liu, L. Zhang, Z. Liu, R. Lin, K. Xiao, X. Luo, J. Shi, J. Du, F. Meng, L. Li, Z. Liu and H. Tan, Sci China Chem, 2021, 64, 2025–2034.

171 H. Gao, Q. Lu, K. Xiao, Q. Han, R. Lin, Z. Liu, H. Li, L. Li, X. Luo, Y. Gao, Y. Wang, J. Wen, Z. Zou, Y. Zhou and H. Tan, Solar RRL, , DOI:10.1002/solr.202100814.

172 J. H. Heo and S. H. Im, Advanced Materials, 2016, 28, 5121–5125.

175 R. Sheng, M. T. Hörantner, Z. Wang, Y. Jiang, W. Zhang, A. Agosti, S. Huang, X. Hao, A. Ho-Baillie, M. Green and H. J. Snaith, Journal of Physical Chemistry C, 2017, 121, 27256–27262.

177 C. Li, Z. S. Wang, H. L. Zhu, D. Zhang, J. Cheng, H. Lin, D. Ouyang and W. C. H. Choy, Adv Energy Mater, , DOI:10.1002/aenm.201801954.

178 Z. Yu, Z. Yang, Z. Ni, Y. Shao, B. Chen, Y. Lin, H. Wei, Z. J. Yu, Z. Holman and J. Huang, Nat Energy, 2020, 5, 657–665.

179 D. Zhao, C. Wang, Z. Song, Y. Yu, C. Chen, X. Zhao, K. Zhu and Y. Yan, ACS Energy Lett, 2018, 3, 305–306.

181 A. Rajagopal, Z. Yang, S. B. Jo, I. L. Braly, P. W. Liang, H. W. Hillhouse and A. K. Y. Jen, Advanced Materials, , DOI:10.1002/adma.201702140.

182 Y.-H. Chiang, K. Frohna, H. Salway, A. Abfalterer, B. Roose, M. Anaya and S. D. Stranks, Efficient all-perovskite tandem solar cells by dual-interface optimisation of vacuum-deposited wide-bandgap perovskite, .

183 D. Vidyasagar, Y. Yun, J. Yu Cho, H. Lee, K. Won Kim, Y. Tae Kim, S. Woong Yang, J. Jung, W. Chang Choi, S. Kim, R. Kumar Gunasekaran, S. B. Kang, K. Heo, D. H. Kim, J. Heo and S. Lee, Journal of Energy Chemistry, 2024, 88, 317–326.

184 L. Qiao, T. Ye, P. Wang, T. Wang, L. Zhang, R. Sun, W. Kong and X. Yang, Adv Funct Mater, , DOI:10.1002/adfm.202308908.

186 H. Guan, S. Zhou, S. Fu, D. Pu, X. Chen, Y. Ge, S. Wang, C. Wang, H. Cui, J. Liang, X. Hu, W. Meng, G. Fang and W. Ke, Advanced Materials, , DOI:10.1002/adma.202307987.

187 T. Ma, H. Wang, Z. Wu, Y. Zhao, C. Chen, X. Yin, L. Hu, F. Yao, Q. Lin, S. Wang, D. Zhao, X. Li and C. Wang, Advanced Materials, , DOI:10.1002/adma.202308240.

188 H. Sun, K. Xiao, H. Gao, C. Duan, S. Zhao, J. Wen, Y. Wang, R. Lin, X. Zheng, H. Luo, C. Liu, P. Wu, W. Kong, Z. Liu, L. Li and H. Tan, Advanced Materials, , DOI:10.1002/adma.202308706.

189 S. Lee, M. Y. Woo, C. Kim, K. W. Kim, H. Lee, S. B. Kang, J. M. Im, M. J. Jeong, Y. Hong, J. W. Yoon, S. Y. Kim, K. Heo, K. Zhu, J. S. Park, J. H. Noh and D. H. Kim, Chemical Engineering Journal, , DOI:10.1016/j.cej.2023.147587.

190 F. Yang, P. Tockhorn, A. Musiienko, F. Lang, D. Menzel, R. Macqueen, E. Köhnen, K. Xu, S. Mariotti, D. Mantione, L. Merten, A. Hinderhofer, B. Li, D. R. Wargulski, S. P. Harvey, J. Zhang, F. Scheler, S. Berwig, M. Roß, J. Thiesbrummel, A. Al-Ashouri, K. O. Brinkmann, T. Riedl, F. Schreiber, D. Abou-Ras, H. Snaith, D. Neher, L. Korte, M. Stolterfoht and S. Albrecht, Advanced Materials, , DOI:10.1002/adma.202307743.

192 S. Li, Z. Zheng, J. Ju, S. Cheng, F. Chen, Z. Xue, L. Ma and Z. Wang, Advanced Materials, , DOI:10.1002/adma.202307701.

193 D. Yu, M. Pan, G. Liu, X. Jiang, X. Wen, W. Li, S. Chen, W. Zhou, H. Wang, Y. Lu, M. Ma, Z. Zang, P. Cheng, Q. Ji, F. Zheng and Z. Ning, Nat Energy, , DOI:10.1038/s41560-023-01441-2.

194 D. Pu, S. Zhou, H. Guan, P. Jia, G. Chen, H. Fang, S. Fu, C. Wang, H. Hushvaktov, A. Jumabaev, W. Meng, X. Wang, G. Fang and W. Ke, Adv Funct Mater, , DOI:10.1002/adfm.202314349.

195 Z. Xie, S. Chen, Y. Pei, L. Li, S. Zhang and P. Wu, Chemical Engineering Journal, , DOI:10.1016/j.cej.2024.148638.

196 A. Maxwell, H. Chen, L. Grater, C. Li, S. Teale, J. Wang, L. Zeng, Z. Wang, S. M. Park, M. Vafaie, S. Sidhik, I. W. Metcalf, Y. Liu, A. D. Mohite, B. Chen and E. H. Sargent, ACS Energy Lett, 2024, 9, 520–527.

198 X. Lv, W. Li, J. Zhang, Y. Yang, X. Jia, Y. Ji, Q. Lin, W. Huang, T. Bu, Z. Ren, C. Yao, F. Huang, Y. B. Cheng and J. Tong, Journal of Energy Chemistry, 2024, 93, 64–70.

199 X. Jiang, Q. Zhou, Y. Lu, H. Liang, W. Li, Q. Wei, M. Pan, X. Wen, X. Wang, W. Zhou, D. Yu, H. Wang, N. Yin, H. Chen, H. Li, T. Pan, M. Ma, G. Liu, W. Zhou, Z. Su, Q. Chen, F. Fan, F. Zheng, X. Gao, Q. Ji and Z. Ning, Natl Sci Rev, , DOI:10.1093/nsr/nwae055.

200 Q. Sun, Z. Zhang, H. Yu, J. Huang, X. Li, L. Dai, Q. Wang, Y. Shen and M. Wang, Energy Environ Sci, , DOI:10.1039/d3ee03898j.

201 P. Jia, G. Chen, G. Li, J. Liang, H. Guan, C. Wang, D. Pu, Y. Ge, X. Hu, H. Cui, S. Du, C. Liang, J. Liao, G. Xing, W. Ke and G. Fang, Advanced Materials, , DOI:10.1002/adma.202400105.

202 J. Zhou, T. Wen, J. Sun, Z. Shi, C. Zou, Z. Shen, Y. Li, Y. Wang, Y. Lin, S. Yang, F. Liu and Z. Yang, ACS Energy Lett, 2024, 1984–1992.

203 Y. Dong, R. Yu, G. Su, Z. Ma, Z. He, R. Wang, Y. Zhang, J. Yang, Y. Gong, M. Li and Z. Tan, Advanced Materials, , DOI:10.1002/adma.202312704.

204 X. Hu, F. Yao, C. Wang, H. Cui, P. Jia, S. Du, S. Zhou, H. Guan, Q. Lin, W. Ke, C. Tao and G. Fang, Chemical Engineering Journal, 2024, 151379.
